# Identification of nucleobase chemical modifications that reduce the hepatotoxicity of gapmer antisense oligonucleotides

**DOI:** 10.1093/nar/gkac562

**Published:** 2022-07-08

**Authors:** Tokuyuki Yoshida, Kunihiko Morihiro, Yuki Naito, Atsushi Mikami, Yuuya Kasahara, Takao Inoue, Satoshi Obika

**Affiliations:** Division of Molecular Target and Gene Therapy Products, National Institute of Health Sciences, Kawasaki, Kanagawa, Japan; Graduate School of Pharmaceutical Sciences, Osaka University, Suita, Osaka, Japan; National Institutes of Biomedical Innovation, Health and Nutrition (NIBIOHN), Ibaraki, Osaka, Japan; Database Center for Life Science (DBCLS), 1111 Yata, Mishima, Shizuoka 411-8540, Japan; National Institute of Genetics, 1111 Yata, Mishima, Shizuoka 411-8540, Japan; Graduate School of Pharmaceutical Sciences, Osaka University, Suita, Osaka, Japan; Graduate School of Pharmaceutical Sciences, Osaka University, Suita, Osaka, Japan; National Institutes of Biomedical Innovation, Health and Nutrition (NIBIOHN), Ibaraki, Osaka, Japan; Division of Molecular Target and Gene Therapy Products, National Institute of Health Sciences, Kawasaki, Kanagawa, Japan; Graduate School of Pharmaceutical Sciences, Osaka University, Suita, Osaka, Japan; National Institutes of Biomedical Innovation, Health and Nutrition (NIBIOHN), Ibaraki, Osaka, Japan

## Abstract

Currently, gapmer antisense oligonucleotide (ASO) therapeutics are under clinical development for the treatment of various diseases, including previously intractable human disorders; however, they have the potential to induce hepatotoxicity. Although several groups have reported the reduced hepatotoxicity of gapmer ASOs following chemical modifications of sugar residues or internucleotide linkages, only few studies have described nucleobase modifications to reduce hepatotoxicity. In this study, we introduced single or multiple combinations of 17 nucleobase derivatives, including four novel derivatives, into hepatotoxic locked nucleic acid gapmer ASOs and examined their effects on hepatotoxicity. The results demonstrated successful identification of chemical modifications that strongly reduced the hepatotoxicity of gapmer ASOs. This approach expands the ability to design gapmer ASOs with optimal therapeutic profiles.

## INTRODUCTION

Gapmer antisense oligonucleotides (ASOs) containing chemically-modified ribose with a high binding affinity to RNA in the wing regions and natural deoxyribonucleotides in the central gap region are promising therapeutic agents (1). Gapmer ASOs bind to target RNA in a sequence-specific manner to form ASO/RNA heteroduplexes, which are recognized by RNase H and the target RNA cleaved. Currently, three 2′-methoxyethyl gapmer ASO therapeutics have been approved [mipomersen (Kynamro) (2), inotersen (Tegsedi) (3) and volanesorsen (Waylivra) (4)], with many other gapmer ASO candidates currently in clinical trials (1). Phosphorothioate (PS) linkages are chemical modifications that enhance *in vivo* stability and used in most gapmers. Additionally, locked nucleic acids (LNAs) and their analogs have garnered attention as gapmer ASO modifications owing to their high RNA-binding affinity (5,6).

Gapmer ASOs are important drug candidates, but they have the potential to induce hepatotoxicity. Several groups have reported that sequence motifs can enhance hepatotoxicity (7,8). Hagedorn *et al.* (8) reported 17 dinucleotide motifs capable of influencing hepatotoxicity when present in ASO sequences using machine learning approaches. Burdick et al. (7) reported that TGC/TCC motifs, as consensus sequences, associated with the hepatotoxic potential of ASO. Gapmer ASO-mediated hepatotoxic effects can be classified as hybridization-dependent and -independent (Supplementary Figure S1: conceptual diagram). Hybridization-dependent effects are potentially caused by inadvertent binding of ASOs to RNA with sequences similar to that of the target RNA. Recent studies showed that the hepatotoxicity of gapmer ASOs is mediated by RNase H-dependent reduction of unintended off-target RNAs (9–11). By contrast, ASO toxicity via hybridization-independent effects is assumed to be due to interactions between ASOs and cellular proteins and unrelated to the Watson–Crick base pairing of ASOs to RNA (12–15). Previous studies have revealed RNase H1-dependent delocalization of paraspeckle proteins to nucleoli as an early event in gapmer ASO-mediated hepatotoxicity, followed by nucleolar stress, p53 activation, and apoptotic cell death (13–15).

Hepatotoxicity of gapmer ASOs can be reduced, and modulating the melting temperature (*T*_m_) by changing the binding affinity between ASOs and complementary RNAs is one of the strategies to achieve this (16–18). Other studies report reducing gapmer ASO-specific hepatotoxicity via chemical modifications of sugar residues (13,19–23) or internucleotide linkages (12,24–26). Additionally, we previously showed that replacement of LNAs with an amino-bridged nucleic acid in the wing region of an LNA gapmer reduces hepatotoxicity (21). However, few studies have evaluated nucleobase modifications in terms of reduced hepatotoxicity. Some nucleobase derivatives, such as 5-methylcytosine and N1-methylpseudouracil, were reported to reduce the immune activation of nucleic acid-based drugs without spoiling the therapeutic effects by altering the interaction mode between nucleic acid molecules and immune-related biomolecules (27,28). Such findings prompted us to assume that nucleobase modification should be a promising strategy to reduce the hepatotoxicity by altering the interaction of gapmer ASOs and hepatocellular proteins.

In this study, we focused on the ability of nucleobase modifications to reduce the hepatotoxicity of gapmer ASOs. As TGC/TCC motifs are hepatotoxicity-associated consensus sequences present in LNA gapmer ASOs (7), we examined 17 nucleobase derivatives for the cytosine, thymine, or guanine bases, as well as four novel nucleobase modifications. We subsequently introduced these derivatives into hepatotoxic LNA ASO gapmers and examined their effects on the hepatotoxicity. Our study outcomes led to the identification of several modifications that caused notable reductions in ASO hepatotoxicity.

## MATERIALS AND METHODS

### Antisense oligonucleotides

The protocols used for the chemical synthesis of G4-, G5-, G6- and G7-phosphoramidites are included in the Supplementary Materials. Other modified phosphoramidites were purchased from Glen Research (Sterling, VA, USA). All LNA gapmer ASOs used in this study were synthesized by Gene Design, Inc. (Osaka, Japan). Detailed sequences are shown in Supplementary Tables S1 and S2. The *T*_m_ of the duplexes formed between the LNA gapmer ASOs used in hepatotoxicity screening and RNA complements were estimated using the ‘LNA oligo *T*_m_ prediction tool’ software developed by Exiqon (QIAGEN, Hilden, Germany).

### *In silico* analysis

The 14-mer sequences were randomly designed, and their potential complementary regions were searched. Sequence searches against mouse mRNA (NCBI RefSeq release 60) allowing mismatches were performed using GGGenome (https://GGGenome.dbcls.jp/), which quickly searches short nucleotide sequences utilizing suffix array and FM-index libraries stored on solid state drives, with this also used to search for the off-target candidate genes of ASOs from our previous studies (29,30). Since a pre-mRNA database for searching off-target candidate genes of ASOs had not been publicly established at the beginning of the present study, we screened hepatotoxic ASOs using ASO sequences harboring no complementary regions with a perfect match or 1-mismatch against mouse mRNAs rather than pre-mRNAs. For the subsequent *in silico* analysis using mouse pre-mRNAs, we used sequences retrieved from the D3G (release 21.01; https://d3g.riken.jp) that has recently developed. The off-target candidate pre-mRNA sequences were grouped by a complementarity measure termed as ‘distance’ (*d*), which is defined as the total number of mismatches, insertions, or deletions between the ASO and complementary RNA sequences, as described previously (30).

### Animal experiments

All animal experiments were performed in accordance with the animal welfare bylaws of Shin Nippon Biomedical Laboratories Ltd. (Kagoshima, Japan), which is fully accredited by the Association for Assessment and Accreditation of Laboratory Animal Care International. The experimental procedures were reviewed and approved by the Institutional Animal Care and Use Committee. C57BL/6J mice (male, 5-weeks old) were purchased from Charles River Laboratories Japan, Inc. (Yokohama, Japan) and housed in a ventilated animal room maintained at between 19°C and 25°C under a 12-/12-h light/dark cycle with food and water supplied *ad libitum*. LNA gapmer ASOs (20 mg/kg), TS1-T4-5 or TS1-G8-6 (10 mg/kg), or saline (control) was intravenously administered to the mice, with the injected volume subsequently adjusted to 10 ml/kg. At 96-h post-injection, the mice were anesthetized with 2–4% isoflurane, and blood samples were collected from the posterior vena cava, processed, and the serum was used to analyze alanine aminotransferase (ALT) and aspartate aminotransferase (AST) levels. Serum AST and ALT levels were measured using a biochemical autoanalyzer (JCA-BM6070; Japan Electronics Industry Ltd., Osaka, Japan). Liver specimens were also isolated from the euthanized mice, with ∼100 mg of liver being immediately frozen in liquid nitrogen and stored at <−70°C until quantitative reverse transcription polymerase chain reaction (qRT-PCR) analysis.

### RNA purification and qRT-PCR

Mouse liver specimens were homogenized using a Shake Master Auto system (Bio Medical Science, Inc., Tokyo, Japan), and total RNA was isolated from the homogenized liver tissues using TRIzol Reagent (Thermo Fisher Scientific, Waltham, MA, USA) according to manufacturer instructions. RNA was reverse transcribed and used as the template for qRT-PCR, which was performed using the One Step TB Green PrimeScript RT-PCR kit (TaKaRa Bio, Inc., Shiga, Japan) and analyzed using the 7500 Fast Real-Time PCR system (Applied Biosystems, Foster City, CA, USA). The primers used are shown in Supplementary Table S3. The level of target gene expression was normalized to that of mouse *glyceraldehyde 3phosphate dehydrogenase* expression.

### *T*_m_ measurements

UV melting experiments were carried out using a UV-1650PC and UV-1800 spectrophotometer (SHIMADZU) equipped with a *T*_m_ analysis accessory. Equimolar amounts of two single-stranded oligonucleotides were dissolved in 10 mM sodium phosphate buffer (pH 7.2) containing 100 mM NaCl to give a final strand concentration of 4.0 μM. The mixture was annealed by heating at 90°C followed by slow cooling to room temperature. The melting profile was recorded at 260 nm in the forward and reverse direction from 20°C to 90°C at a scan rate of 0.5°C/min. All UV melting assays were performed at least three times, and *T*_m_ values were calculated by the average method of melting curves.

### Statistical analysis

Statistical analyses were performed using Ekuseru-Toukei software (Social Survey Research Information Co., Ltd., Tokyo, Japan). All data are presented as the mean ± standard error, and a *P* < 0.05 was considered significant. Serum AST and ALT levels, and on-target activities were compared among groups using analysis of variance, with differences compared using Tukey's test.

## RESULTS

### Screening of LNA gapmer ASOs that induce hepatotoxicity

We first performed *in silico* analysis against mouse RNA databases using GGGenome. We obtained a total of 7183 14-mer sequences having no complementary regions with a perfect match or 1-mismatch against mouse mRNAs. We selected 149 of these sequences, many of which harbored TGC and/or TCC motifs reportedly involved in hepatotoxicity (Supplementary Table S1) (7). We then synthesized 149 LNA gapmers based on the selected sequences and performed hepatotoxicity screening *in vivo*. We identified five gapmers that substantially increased the serum AST/ALT level to >100 U/l and named these TS1-ASO, TS2-ASO, TS3-ASO, TS4-ASO and TS5-ASO (Table 1 and Supplementary Table S1). Additionally, we predicted the *T*_m_ values of the duplexes formed between the 149 LNA gapmers and their complementary ssRNA targets (Supplementary Figure S2) (17) but did not find any correlation between hepatotoxicity and the *T*_m_ values.

**Table 1.** TS-ASO sequences obtained from the hepatotoxicity screening^a^

		Number of motifs			
Name	Sequence	TGC	TCC	AST (Ul^–1^)	ALT (Ul^–1^)
TS1-ASO	GTTATGCCACCCTA	1	0	9437	15 602
TS2-ASO	GTCCGCATGCCTAA	1	1	1364	1617
TS3-ASO	GATATGCCCTACTA	1	0	448	1061
TS4-ASO	GTATGCCTCCGTTA	1	1	2164	600
TS5-ASO	GCTATGTTAGTCCG	0	1	287	291

^a^All oligonucleotides were fully modified with phosphorothioate linkages. Gap DNA and LNA are indicated in black and green, respectively. C57BL/6J mice (*n* = 2 animals per group) were intravenously injected with a dose of 20 mg/kg. Ninety-six hours post injection, serum aspartate aminotransferase (AST)/alanine aminotransferase (ALT) levels were determined.

### Identification of potential nucleobase modifications that reduce LNA gapmer hepatotoxicity

**Figure 1.**
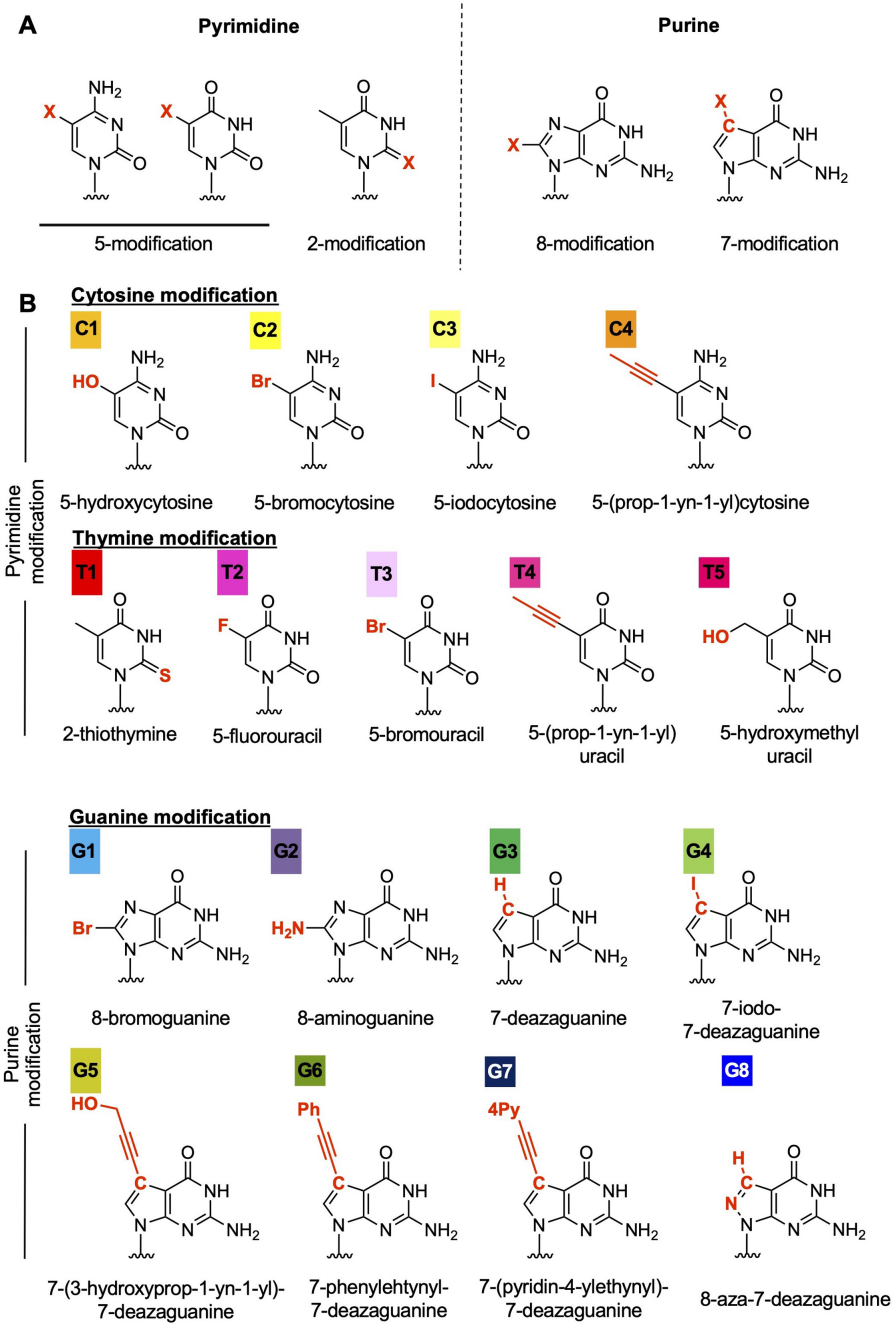
Nucleobase modification strategy to reduce LNA gapmer hepatotoxicity. (**A**) Position of chemical modification of the nucleobase. The 5- or 2-position of pyrimidine and 8- or 7-position of purine were selected for chemical modifications. (**B**) Nucleobase derivatives used in the study. Each color indicates a different nucleobase modification.

**Figure 2.**
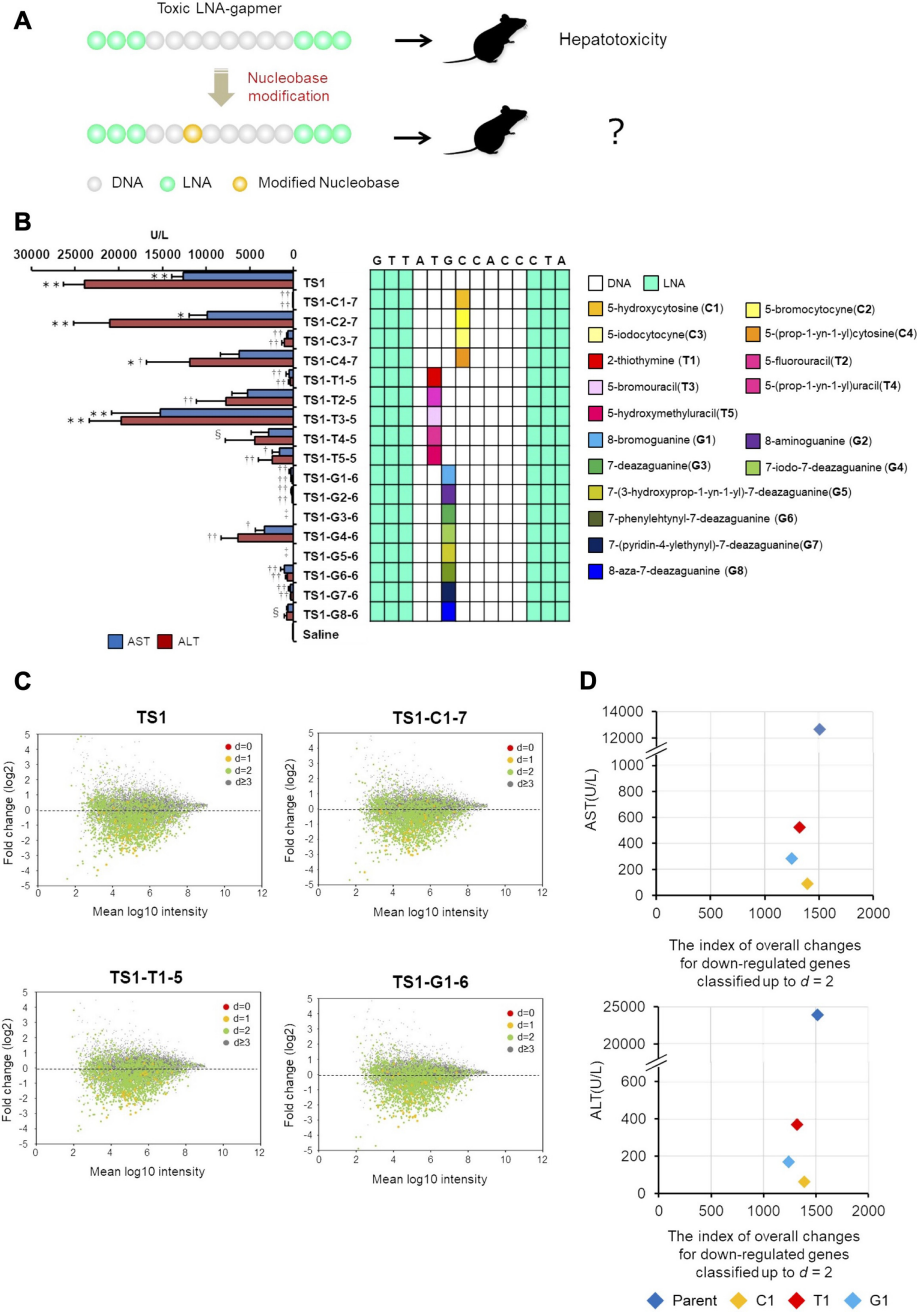
Effects of nucleobase modification on TS1-ASO hepatotoxicity and gene expression. (**A**) Introduction of a modified nucleobase on the TS-ASO. A cytosine, thymine, or guanine nucleobase in the gap region was replaced with a chemically modified analog. (**B**) Hepatotoxicity of the TS1-ASO series. Left panel: Serum AST and ALT levels. C57BL/6J mice were intravenously injected 10 mg/kg TS1-T4-5 or TS1-G8-6, or 20 mg/kg other TS1-ASO series. Ninety-six hours post injection, serum AST and ALT levels were measured. ^‡^The mice were euthanized due to severe physical toxicity within 96 h post administration. ^§^The mice were intravenously injected at a dose of 10 mg/kg. Results are expressed as mean ± SE (*n* = 4). Asterisks indicate a significant difference compared with the control group (**P < 0.01, *P < 0.05). Daggers indicate a significant difference compared with the TS1-ASO-treated group (^††^*P* < 0.01, ^†^*P* < 0.05). Right panel: Schematic illustration of the parent TS1-ASO and TS1-ASO analogs with nucleobase modifications in the gap region. (**C**) Scatter plots from microarray analysis of the cells treated with TS1-ASO, TS1-C1-7, TS1-T1-5, and TS1-G1-6. The multiplied fluorescence intensity of control group is shown on the horizontal axis, and the proportion of change in gene expression as the results of introduction of each ASO (expressed logarithmically) is shown on the vertical axis. d: Distance; The total number of mismatches, insertions, or deletions between the ASO and complementary RNA sequences. Red dots: *d*= 0 genes, which have perfect complementarity. Orange dots: *d*= 1 genes. Light green dots: *d*= 2 genes. Gray dots: *d*≧3 genes. (**D**) The relationship between hepatotoxicity (AST and ALT values) and the index of overall down-regulation. The levels of serum AST and ALT in mice treated with TS1-ASO or nucleobase-modified TS1-ASOs are shown on the vertical axis. The index of overall changes for down-regulated genes classified up to *d* = 2 in the cells treated with TS1-ASO or nucleobase-modified TS1-ASOs is shown on the horizontal axis. The index was quantified by taking the logarithm of the ratio of gene expression changes and calculating the sum of the absolute value of it (see a conceptual diagram of the index in Supplementary Figure S4). Blue plot indicates TS1-ASO, and each color indicates each TS1-ASO analogue with nucleobase modification.

To explore strategies to reduce the hepatotoxicity of LNA gapmers, we tested nucleobase modifications in the hepatotoxic gapmers. Considering the effect on base pairing, we selected the 5- or 2-position of pyrimidine and 8- or 7-position of purine for chemical modifications (Figure 1A), resulting in a total of 17 nucleobase derivatives (Figure 1B). We then replaced a cytosine, thymine, or guanine nucleobase in the gap region of the selected LNA gapmers with a chemically-modified analog (Figure 2A), and the gapmers were then administered to mice in order to evaluate their respective hepatotoxicity. We initially introduced a modified nucleobase into the TGC motif of TS1-ASO, which caused the most severe hepatotoxicity in our screening process. Nucleobase modification with C1, C3, T1, G1, G2, G6 or G7 reduced serum AST/ALT levels by 0.3–4.3% as compared with that observed with the parent TS1-ASO (Figure 2B). The TS1-ASO analogs containing G3- or G5-treated mice showed decreased spontaneous movement within 96-h post-administration (Figure 2B). The introduction of C1 to gap positions 8, 10 and 11 reduced serum AST/ALT levels, although the decreased levels of hepatotoxicity were lower than those achieved upon introducing C1 to gap position seven (Supplementary Figure S3A). Similar results were observed for TS2- and TS3-ASO, where modification with C1, G1, G2, G6 or G7 substantially reduced hepatotoxicity as compared with that observed with the parent sequences (Supplementary Figure S3B and C). Except in the case of TS2-T1-8, T1 also reduced hepatotoxicity compared with that observed with the parent TS2 (Supplementary Figure S3B). These observations suggested that the hepatotoxicity of LNA gapmers would generally be reduced as a result of nucleobase modification, although the extent would depend on the specific chemistry and modified position (Supplementary Table S4). We then examined the effects of the nucleobase derivatives introduced into the TS-ASOs on gene expression, and observed no dramatic changes in gene expression (Figure 2C and Supplementary Figure S3D). Furthermore, we analyzed the correlation between changes in gene expression and hepatotoxicity for each TS-ASO (Figure 2D, Supplementary Figures S3E and S4. Supplementary Table S5) and found no correlation between altered gene expression and significantly decreased hepatotoxicity resulting from the nucleobase modifications.

**Figure 3.**
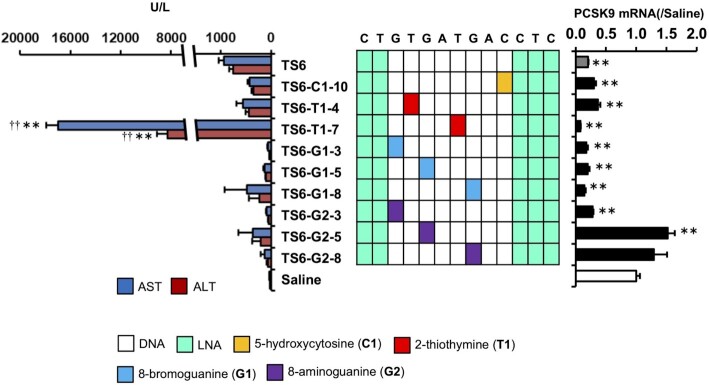
Effects of nucleobase modification on TS6-ASO hepatotoxicity and on-target activity. Hepatotoxicity and on-target activity of TS6-ASO series. Left panel: Serum AST and ALT levels. Middle panel: Schematic of the parent TS6-ASO and TS6-ASO analogs with nucleobase modification in the gap region. Right panel: On-target activity. C57BL/6J mice were intravenously injected 20 mg/kg TS6-ASO series. Ninety-six hours post injection, serum AST and ALT levels and *Pcsk9* mRNA level were measured. Results are expressed as mean ± SE (*n* = 4). Asterisks indicate a significant difference compared with the control group (**P < 0.01). Daggers indicate a significant difference compared with the TS6-ASO-treated group (^††^*P* < 0.01).

To investigate the generality of the nucleobase-modification strategy, we used LNA gapmer ASOs demonstrated as hepatotoxic in our previous study (TS6-ASO, TS7-ASO and TS10-ASO) or other studies (TS8-ASO and TS9-ASO) (31) (Supplementary Table S2). We replaced one of the nucleobases in the gap region of each ASO with a chemically-modified analog of C1, T1, G1, G2, G4, G6 and G7, resulting in a total of 52 ASO derivatives. We then administered each of the ASO derivatives to mice and evaluated changes in serum AST/ALT levels (Supplementary Table S4). Twenty-nine of the 52 ASO derivatives reduced serum AST/ALT levels relative to those observed with the parent ASOs and regardless of the target genes. Ten of the 52 derivatives-treated mice showed decreased spontaneous movement or dead within 96-h post-administration (Figure 3 and Supplementary Figure S5). The on-target activities were not affected by nucleobase modification in most cases, and the modified position did not correlate with the reduced hepatotoxicity. For TS6, TS6-C1-10, TS6-G1-3 and TS6-G2-3, the dose dependency of hepatotoxicity and on-target activity was observed (Supplementary Figure S6). The therapeutic index increased by the nucleobase modification, especially, G1 and G2 (Supplementary Table S6).

### Multiple nucleobase modifications synergistically reduce LNA gapmer ASO hepatotoxicity

**Figure 4.**
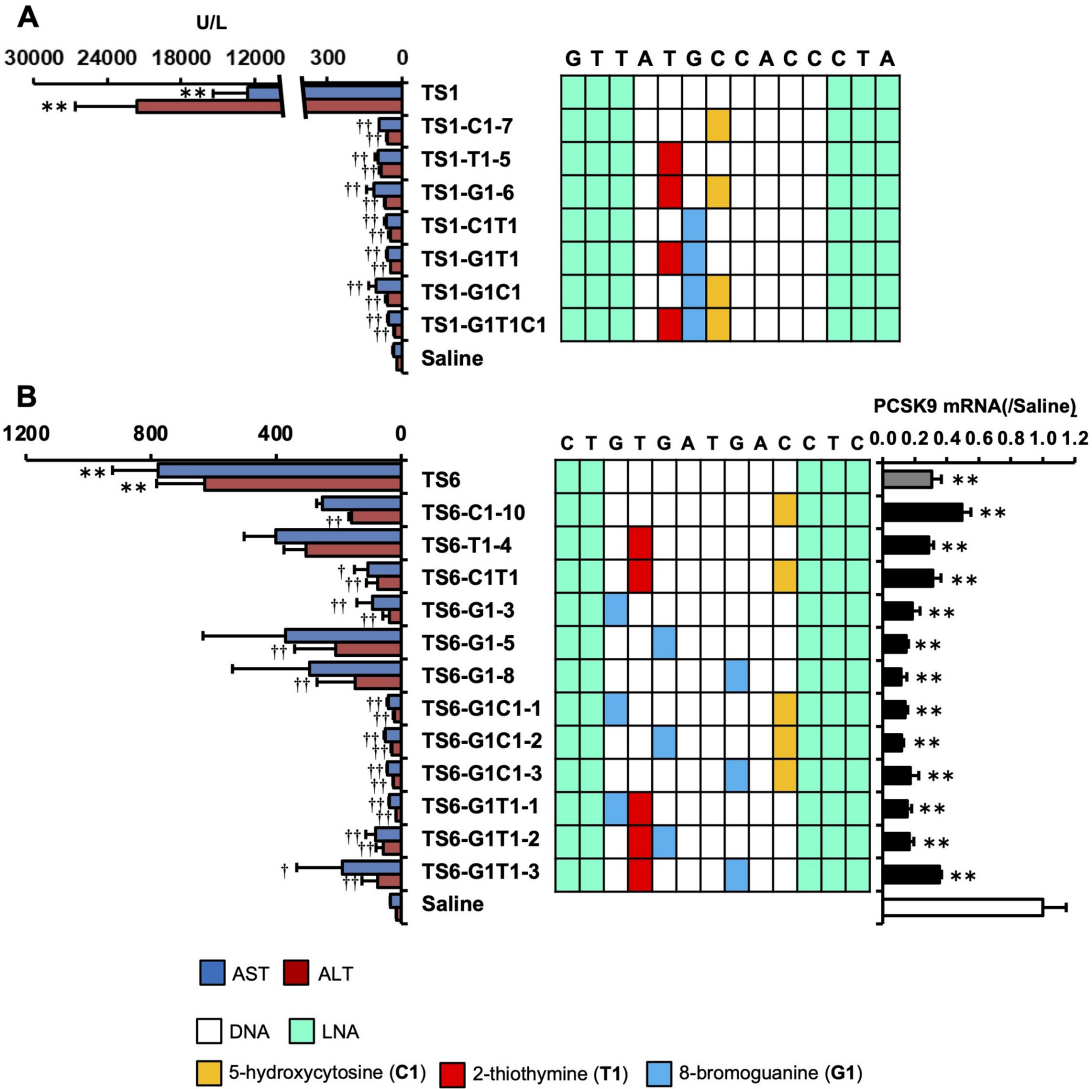
Effect of multiple nucleobase modifications on the hepatotoxicity of LNA gapmer ASOs. (**A**) Hepatotoxicity of the TS1-ASO series. Left panel: Serum AST and ALT levels. Right panel: Schematic of the parent TS1-ASO and TS1-ASO analogs with a single or multiple nucleobase modifications in the gap region. (**B**) Hepatotoxicity and on-target activity of the TS6-ASO series. Left panel: Serum AST and ALT levels. Middle panel: Schematic of the parent TS1-ASO or TS6-ASO analogs with single or multiple nucleobase modifications in the gap region. Right panel: On-target activity. C57BL/6J mice were intravenously injected 20 mg/kg TS1-ASO series or TS6-ASO series. Ninety-six hours post injection, serum AST and ALT levels and *Pcsk9* mRNA level (TS6-ASO target) were measured. Results are expressed as mean ± SE (*n* = 4). Asterisks indicate a significant difference compared with the control group (**P < 0.01). Daggers indicate a significant difference compared with the TS1-ASO-treated or TS6-ASO-treated group (^††^*P* < 0.01, ^†^*P* < 0.05).

We found that even a single nucleobase modification reduced LNA gapmer ASO hepatotoxicity. To further explore the efficacy of this strategy, we investigated multiple replacements of the selected unnatural nucleobases in an ASO. The introduction of C1, T1 and G1 was highly effective at reducing hepatotoxicity in each TS-ASO; they were introduced into TS1-ASO and TS6-ASO at 2–3 sites to investigate their additive effects on reducing hepatotoxicity. Figure 4A shows that TS1 with both C1 and T1 (TS1-C1T1), both G1 and T1 (TS1-G1T1), and both G1 and C1 (TS1-G1C1) reduced AST/ALT levels as compared with a single modification. Moreover, the reduction in hepatotoxicity was further enhanced upon introduction of nucleobase modifications at three sites (TS1-G1T1C1). We then applied additional multiple nucleobase modifications to TS6 (Figure 4B) and observed a similar synergistic reduction in hepatotoxicity. Introduction of G1 alone in TS6-ASO resulted in a varied degree of hepatotoxicity depending on the position into which it was introduced; however, G1 introduction along with C1 or T1 reduced the degree of hepatotoxicity comparably in all TS-ASOs, except for TS6-G1T1-3. Importantly, TS6-ASO did not lose its antisense effects as a result of multiple nucleobase modifications.

### Characterization of TS-ASOs containing nucleobase modifications

**Figure 5.**
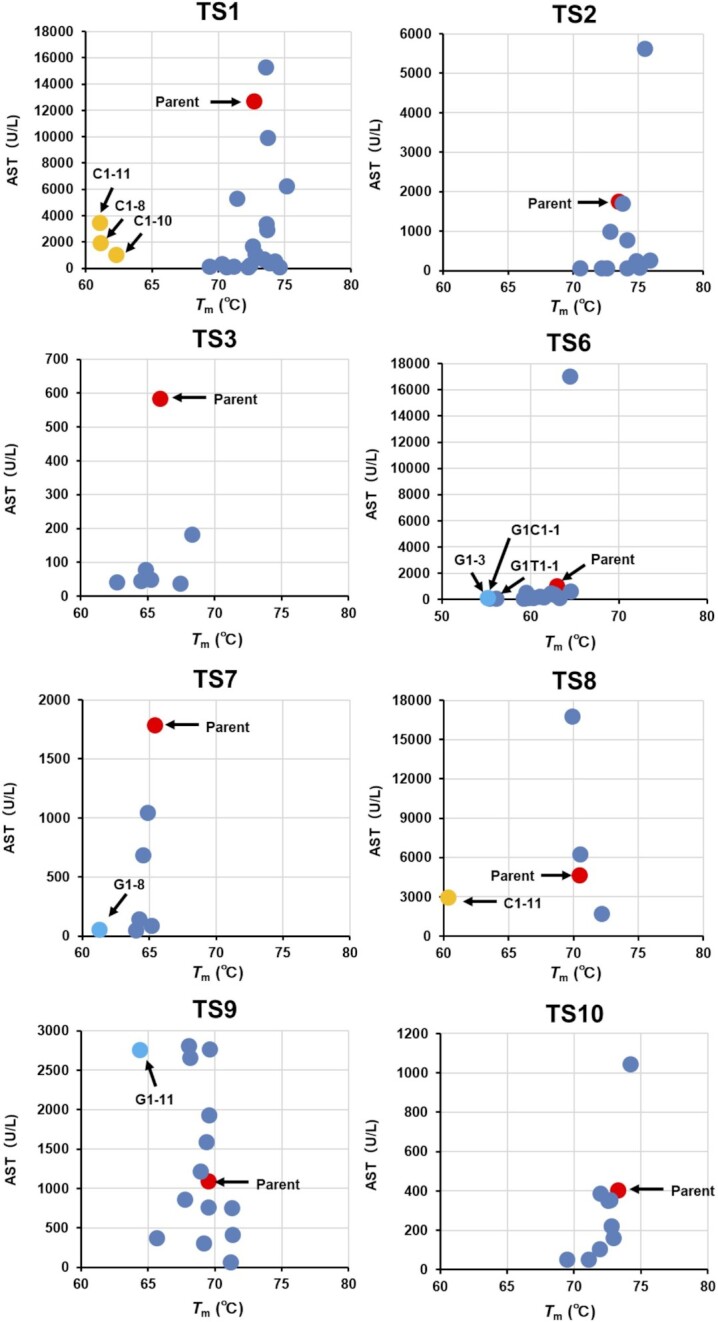
The relationship between hepatotoxicity (AST values) and *T*_m_ values of TS-ASOs or nucleobase-modified TS-ASO. The serum AST levels are shown on the horizontal axis and the *T*_m_ values of the duplexes formed between TS-ASOs or nucleobase-modified TS-ASOs and their complementary RNAs are shown on the vertical axis. The red dots indicate TS-ASOs. The blue dots indicate nucleobase-modified TS-ASOs. The arrows indicate nucleobase-modified TS-ASO which decreased the *T*_m_ values by >5°C compared with the parent TS-ASO.

To characterize the TS-ASOs harboring nucleobase derivatives, we measured the *T*_m_ values of the duplexes formed between TS-ASOs or nucleobase-modified TS-ASOs and their complementary RNAs (Supplementary Table S7). Among 100 TS-ASOs with nucleobase derivatives, nine TS-ASOs with C1 or G1, which reportedly destabilize the duplexes, decreased the *T*_m_ values by >5°C compared with the parent sequence (32,33). The *T*_m_ values for most of the other modified TS-ASOs were equivalent to those of the parent ASOs (Δ*T*_m_ values were within ±2°C). As shown in Figure 5, no obvious correlation was observed between the *T*_m_ value and serum AST/ALT levels for each modified TS-ASO. These results suggested that the decrease in hepatotoxicity following introduction of nucleobase derivatives was unlikely to be due to changes in the binding affinity of the LNA gapmer ASOs to complementary RNAs.

## DISCUSSION

We found that among the 149 LNA gapmer ASOs harboring reported sequences associated with hepatotoxicity, only five (∼3%) resulted in elevated serum AST/ALT levels (Table 1 and Supplementary Table S1). By contrast, Hagedorn *et al.* (8) reported that ∼50% of LNA gapmers caused hepatotoxicity under their reported experimental conditions. Although it is difficult to simply compare the results between studies due to differences in dosing methods, the percentage of LNA gapmers resulting in hepatotoxicity may be small if their sequences avoid the effects of hybridization with mRNA. Recent studies suggest a correlation between target RNA-binding affinity and the hybridization-dependent hepatotoxicity of ASOs (16,17,34); however, we found no correlation between hepatotoxicity and predicted *T*_m_ values for the 149 LNA gapmers screened in this study (Supplementary Figure S2). Additionally, no clear correlation was observed between reduced hepatotoxicity and the changes in *T*_m_ values resulting from the introduction of nucleobase modifications (Figure 5). To analyze how the nucleobase modifications affect the gene expression of gapmer ASOs, the changes in gene expression were quantified and compared with those in hepatotoxicity (Figure 2 and Supplementary Figure S3, Table S5). The index of overall changes for down-regulated genes classified up to *d* = 2 for TS1-C1, TS1-T1, and TS1-G1 as a ratio of parent-TS1 is 91.8, 87.1 and 82.2%, respectively. On the other hand, the ratio of hepatotoxic markers observed with each modified TS1-ASO as a ratio of parent-TS1 are 0.7, 4.1 and 2.2% for AST and 0.3, 1.5 and 0.7% for ALT, respectively. A similar trend was observed for TS2-ASO and TS3-ASO. This means that there is no clear correlation between the changes in the off-target effects and the hepatotoxicity observed with the introduction of nucleobase modification. Based on these results, we inferred that hybridization with complementary RNAs does not strongly affect the decreased hepatotoxicity of gapmer ASOs. As mentioned above, gapmer ASO-mediated hepatotoxic effects can be classified conceptually as hybridization-dependent and -independent (Supplementary Figure S1). We speculated that the nucleobase modifications used in the present study mainly reduced the hybridization-independent toxicity of the LNA gapmers rather than the hybridization-dependent toxicity.

Here, we identified nucleobase derivatives capable of reducing the hepatotoxicity of LNA gapmer ASOs. Among the 17 nucleobase derivatives, C1 (5-hydroxycytosine), T1 (2-thiothymine), and G1 (8-bromoguanine) considerably reduced the hepatotoxicity of the LNA gapmers. C1 is a product of DNA oxidation (35), but an LNA gapmer containing three C1 residues exhibits a high antisense effect against hepatitis C virus RNA (36). T1 has been used to avoid forming wobble-type T:G mismatch base pairs (37); however, the replacement of the oxygen atom at the 2-position to a larger and more hydrophobic sulfur atom affects the interaction of the ASOs with proteins. In fact, a report showed that the T1 nucleotide demonstrates a higher recognition capacity by polymerase than natural thymidine (38). Additionally, G1 prefers a non-canonical syn conformation due to steric repulsion between the C8-bromine atom and the 4′-oxygen atom in the anti-isomer, possibly resulting in changes in the higher-order structures of ASOs (39). Recently, investigators at Ionis Pharmaceuticals and their collaborators performed X-ray crystallography of a PS-modified ASO complexed with the transcription factor positive cofactor 4 (PC4), demonstrating the feasibility of various interactions between ASO nucleobases and proteins (40). Additionally, they found that substitution of specific nucleotides in the ASOs with abasic nucleotides significantly altered their affinity for PC4 (41). These findings suggest that the observed reductions in hepatotoxicity by the nucleobase-modified LNA gapmers in the present study may have been due to altered interactions between the LNA gapmers and unknown proteins involved in hepatotoxicity according to each derivative.

Burdick *et al.* (7) showed that ASOs harboring TGC/TCC motifs were more likely to bind to hepatocellular proteins and induce hepatotoxicity. However, most of the gapmers harboring TGC and/or TCC motifs designed in the present study (89 of 94 gapmers) did not induce hepatotoxicity (Supplementary Table S1), suggesting that the presence of these motifs does not necessarily contribute to hepatotoxicity in the absence of complementarity to any mRNAs. Other studies also proposed that hepatotoxicity can be induced by interaction of gapmer ASO with some proteins (13–15). Although studies have shown that hepatotoxicity can be reduced by chemical modifications of sugar residues or internucleotide linkages, the number of effective modification sites was limited. For sugar modifications, single 2′-O-methyl modification at gap position two from the 5′-side reduced protein binding and substantially decreased ASO hepatotoxicity (13). Furthermore, replacing one PS linkage with alkylphosphonates at gap position two or three from the 5′-side reduces ASO hepatotoxicity (12). Additionally, chemical modification at the 5′-side strongly affect the interaction between proteins and the modified gapmer ASO (42). In the present study, we found that the application of the nucleobase-modification technology was not limited at a particular site of ASOs, and multiple modifications could decrease ASO hepatotoxicity synergistically. However, further exploratory studies are warranted since the mechanism underlying the observed reduction in hepatotoxicity following nucleobase modifications might differ from those reported previously, and specifically those involving introduction of sugar or linkage modifications near the 5′-side.

Based on the above, we propose a designing principle for gapmer ASOs to avoid hepatotoxicity. To reduce hybridization-dependent as well as -independent toxicity, multiple approaches can be combined. For example, toxicity via hybridization-dependent effects can be avoided by optimizing the sequences of gapmer ASOs using *in silico* analysis. Moreover, we previously showed that hybridization-dependent off-target effects can be reduced by appropriately regulating the length of ASOs (43). In the present study, introduction of modified nucleobases effectively reduced the hepatotoxicity of gapmer ASOs possibly via hybridization-independent effects. In particular, C1, G1 and G2 were particularly promising, with T1 recommended for introduction depending on the sequence. Furthermore, the results suggested no limitations on the target position for modification, and that multiple modifications would be more effective. Of the 17 nucleobase derivatives investigated in this study, 13 containing C1, G1, G2 and T1 are commercially available. These results support the concept that chemical modification of nucleobases is effective at reducing the toxicity of not only ASOs but also other nucleic acid-based drug candidates, such as splice-switching oligonucleotides and anti-microRNA oligonucleotides. This technology could further contribute to the development of oligonucleotide therapeutics and serve as a useful tool to elucidate the mechanisms associated with gapmer hepatotoxicity.

## DATA AVAILABILITY

All data are available in the main text or the supplementary materials. The microarray data reported in this paper have been deposited in the Gene Expression Omnibus (GEO) database, www.ncbi.nlm.nih.gov/geo (accession number: GSE197166).

## Supplementary Material

gkac562_Supplemental_File

## Notes

Present address: Kunihiko Morihiro, Department of Chemistry and Biotechnology, Graduate School of Engineering, The University of Tokyo, Bunkyo-ku, Tokyo, Japan.
