# Supplementary material for: Identification of nucleobase chemical modifications that reduce the hepatotoxicity of gapmer antisense oligonucleotides
Source: Nucleic Acids Res. 2022 Jul 8;50(13):7224–34. doi: 10.1093/nar/gkac562 (PMC9303313; doi:10.1093/nar/gkac562)
Supplement: gkac562_Supplemental_File [file gkac562_supplemental_file.pdf]

[Supplementary Data]

Identification of nucleobase chemical modifications that reduce the hepatotoxicity of gapmer antisense oligonucleotides

Tokuyuki Yoshida[1†], Kunihiko Morihiro[2,3,†], Yuki Naito[4,5], Atsushi Mikami[2], Yuuya Kasahara[2,3], Takao Inoue[1*], and Satoshi Obika[2,3,*]

[1] Division of Molecular Target and Gene Therapy Products, National Institute of Health Sciences, Kawasaki, Kanagawa, Japan

[2] Graduate School of Pharmaceutical Sciences, Osaka University, Suita, Osaka, Japan

[3] National Institutes of Biomedical Innovation Health and Nutrition (NIBIOHN), Ibaraki, Osaka, Japan

[4] Database Center for Life Science (DBCLS), 1111 Yata, Mishima, Shizuoka 411-8540, Japan

[5] National Institute of Genetics, 1111 Yata, Mishima, Shizuoka 411-8540, Japan

* To whom correspondence should be addressed. Tel +81-6-6879-8200; Fax: +81-6-6879 -8204; Email: obika@phs.osaka-u.ac.jp (SO)

Correspondence may also be addressed to Takao Inoue: Tel: +81-44-270-6533; Fax: +81-44-270-6539; Email: takao@nihs.go.jp (TI)

† Joint Authors.

Present Address: Kunihiko Morihiro, Department of Chemistry and Biotechnology, Graduate School of Engineering, The University of Tokyo, Bunkyo-ku, Tokyo, Japan

MATERIAL AND METHODS

Cell culture and transfection with ASOs

The mouse hepatic cell line, NMuLi was obtained from the American Type Culture Collection (ATCC; Manassas, VA). The cells were maintained at 37 °C and 5% CO_2 in Dulbecco's Modified Eagle's Medium (Sigma-Aldrich, St. Louis, MO, USA) supplemented with 10% heat-inactivated fetal bovine serum and antibiotics. NMuLi cells were seeded in 12-well plates (Corning; Corning, NY, USA) at 1.5×10^4 cells/well (n = 4/group) and transfected with the ASOs at concentrations of 10 nM using Lipofectamine® 2000 (Invitrogen, Gaithersburg, MD, USA) according to the manufacturer's protocol. Cells treated with transfection reagent in the absence of ASO were used as a control. After a further 24 h, total RNA was isolated using a Qiagen RNeasy Mini Kit (Qiagen, Valencia, CA, USA) according to the manufacturer's instructions.

Microarray analysis

Genome-wide expression analysis was performed using GeneChip™ Mouse Genome 430 2.0 Array (Thermo Fisher Scientific, Waltham, MA, USA) according to the manufacturer's instructions. The scanned data were processed for signal values using Microarray Suite 5.0 software (Affymetrix, CA, USA). Based on the calculated signal value, the ratio of gene expression changes was calculated for all genes in the ASO groups (n=4) compared with the control group (n=4). Genes with sufficient hybridization signals to be called "present" in at least three of four trials were used in this study.

Table S1. Properties of the LNA gapmers used in the hepatotoxicity screening. All oligonucleotides were fully modified with phosphorothioate linkages. Gap DNA and LNA are indicated in black and green font, respectively. C57/BL6 mice (n = 2 animals per group) were intravenously injected at a dose of 20 mg/kg. Ninety-six hours post injection, AST/ALT levels were determined. The RNA melting temperatures (*T*m) of the LNA gapmer ASOs were estimated using the "LNA oligo *T*m prediction tool", a program developed by Exiqon (QIAGEN).

**ID/Name**	**Sequence**	**Number of motifs**		**AST (Ul^{-1})**	**ALT (Ul^{-1})**	**T_m (˚C)**
		**TGC**	**TCC**			
1	GTATTAGCCCCTAG	0	0	52	22	73
2	GCCCTAACTATCAT	0	0	35	17	73
3	GAACCCTAGATCTA	0	0	37	19	66
4	TGGACCAATTAGGC	0	0	53	20	37
5	GTAGGATAGATTAC	0	0	40	12	68
6	CTACTAGTACCCTA	0	0	51	19	58
7	TATTAATCTAGGGC	0	0	30	17	39
8	GGTACACTAACCCC	0	0	36	21	90
9	ACTAACCTTATGGG	0	0	34	17	63
10	GCCCCTATGTTATG	0	0	38	21	71
11	GTATTATCCTGCGC	1	1	34	17	54
**12/TS2**	GTCCGCATGCCTAA	1	1	1364	1617	86
13	CAATTACTCCGTGC	1	1	31	18	44
**14/TS4**	GTATGCCTCCGTTA	1	1	2164	600	74
15	ACTGCGTATATGCC	2	0	155	33	74
16	GAGCGTAGTGCAAC	1	0	47	33	83
17	TGCATACTAGACCG	1	0	44	16	53
18	TGCTAGCTCGTATA	1	0	36	19	54
19	GACCCGGTATGCTA	1	0	40	26	83
20	GCTACGCCTTATGC	1	0	33	19	70
21	TACTATGCGCTAAT	1	0	37	19	70
22	GCCCTTATATGCGG	1	0	31	16	67
23	TAATGCGTATAAGC	1	0	51	29	47
24	CTTAGTGCGACCTA	1	0	38	25	69
25	GCGTATTAGGTTGC	1	0	32	17	46
26	TAACTATTGCCGGC	1	0	43	26	63

Table S1 (Continued).

ID/Name	Sequence	Number of motifs		AST (UI^{-1})	ALT (UI^{-1})	T_m (˚C)
		TGC	TCC			
27	CTAATGCCTAACGT	1	0	42	16	56
28	GTTGTGCGTACCTA	1	0	65	38	77
29	CCTAGTGCGCTATA	1	0	71	39	73
30	GGTTGCGACTTACC	1	0	44	15	86
31	CGAGTATTATGCCC	1	0	54	38	78
32	ATGCCCTACTAGCG	1	0	70	32	57
33	TATGCTAGTACGGC	1	0	35	17	65
34	TACCTAATTGCGAG	1	0	40	23	78
35	TATACTAACCGTCC	0	1	37	23	80
36	CATACTACTATCCG	0	1	39	24	63
37	CGTTAGACTAATCC	0	1	34	17	74
38	GTTAATCCCTAGCG	0	1	46	18	56
39	GTCCGATTACTATG	0	1	56	23	76
40	TACGTTAGATCCCC	0	1	35	16	87
41	ATCCGTCTAACTAA	0	1	46	23	74
42	CGTATGGTTAGTCC	0	1	35	19	80
43	AACTATCGATCCTA	0	1	41	16	66
44	GGATCCTATACGAC	0	1	43	29	82
45	TCCTATACCGACCT	0	1	38	14	92
46	CGTTATATCCTATC	0	1	38	20	64
47	TACGACCTAAGTCC	0	1	33	17	91
**48/TS5**	GCTATGTTAGTCCG	0	1	287	291	83
49	GGGGTCCGCCTATA	0	1	38	24	74
50	ATTAGTCCGTTGGA	0	1	33	16	71
51	ACCGGATCCTAATA	0	1	36	22	80
52	TCGATCCCTATAAG	0	1	34	20	62

Table S1 (Continued).

ID/Name	Sequence	Number of motifs		AST (UI^{-1})	ALT (UI^{-1})	T_m (˚C)
		TGC	TCC			
53	TGCACTATCCCCCC	1	1	48	29	85
54	TATCCTAGCTATCC	0	2	44	16	81
55	CAATCTATGTCCTA	0	1	39	17	51
56	GTATACTATGGTCC	0	1	32	17	86
57	CTAGGTCCTACTAG	0	1	35	19	60
58	CTAGTAGTATCCTA	0	1	40	18	51
59	TAGCACACCAATCC	0	1	39	18	85
60	ATAGTGGCCTATCC	0	1	43	20	86
61	GGGTTAACTATCCA	0	1	34	20	67
62	AATCCCCCTAGGTA	0	1	34	17	76
63	TAAGCTAGGTATCC	0	1	36	19	75
64	GGTACTACTCCTAT	0	1	36	18	73
65	CTAACTACTAGTCC	0	1	35	19	70
66	CTAGCTACTAGTCC	0	1	46	19	80
67	AGTCCCCCCCCTAT	0	1	47	20	103
68	GTCCTAACTTATAC	0	1	35	17	82
69	ATCCTGTAGCTAGC	0	1	35	17	80
70	GACTACCTATCCAC	0	1	36	18	87
71	TAACCTAGATCCTA	0	1	37	14	55
72	TAGTCCAACTAGTA	0	1	37	19	57
73	ATACTCCTACTAGC	0	1	49	30	59
74	ATACCCTTGCTAGG	1	0	43	22	68
75	GTATTGCAAGCTAG	1	0	42	17	70
76	GTCTAGTGCTATAC	1	0	38	23	86
77	GGTTTGATGCACTA	1	0	44	23	64
78	GTGCCTAACCTATC	1	0	37	20	83

Table S1 (Continued).

ID/Name	Sequence	Number of motifs		AST (Ul^{-1})	ALT (Ul^{-1})	T_m (˚C)
		TGC	TCC			
79	GTGCCTTACTATTA	1	0	43	20	69
80	GGTACATGCCCCTA	1	0	51	29	78
81	TAGGTTGAATTGCC	1	0	34	19	68
82	ATAAGTGCCTAGTC	1	0	41	23	75
83	GGTACTACTAATGC	1	0	36	21	58
84	GAATGCTAGCCTAC	1	0	48	22	79
85	GTATTGTATAGTGC	1	0	32	15	48
86	CCTAAACCTAGTGC	1	0	35	18	59
87	GCTACTAGTGTGCC	1	0	36	18	67
88	GTATAGTGCCAATC	1	0	35	16	76
89	CCTAATGGTTATGC	1	0	37	18	59
90	GCTGCCTATTACTA	1	0	49	19	56
91	CTAATTGCCCTAGT	1	0	37	20	67
92	GACCCTATATGCTG	1	0	43	21	77
93	TAATGCACCTACCC	1	0	49	19	77
94	GGCCTAATGCATAT	1	0	31	18	65
95	CACCCATATTATGC	1	0	40	21	64
96	GAGTATGCTAACTC	1	0	32	18	79
97	TTATTGTGCTACCC	1	0	46	23	79
**98/TS3**	GATATGCCCTACTA	1	0	448	1061	71
99	GACTGCCTAATCAT	1	0	51	20	80
100	ACTAGTGCATTACT	1	0	34	17	76
**101/TS1**	GTTATGCCACCCTA	1	0	9437	15602	80
102	TATGCTAGCAGGGC	1	0	46	18	62
103	GATTGCTAACCTTA	1	0	49	26	72
104	CCATACTAGTATGC	1	0	35	18	52

Table S1 (Continued).

ID/Name	Sequence	Number of motifs		AST (Ul^{-1})	ALT (Ul^{-1})	T_m (˚C)
		TGC	TCC			
105	CTATTAGGGGGGT	0	0	30	13	65
106	CCCCCCTATCACTA	0	0	42	18	83
107	CTATACCCCCCTAT	0	0	44	16	69
108	GTACCCCCCTATAC	0	0	33	17	86
109	ACTAGTAGGGTACC	0	0	34	18	86
110	ATTGACCCCCCCTA	0	0	34	18	84
111	GGGATATAGTACCC	0	0	36	22	75
112	GTATTGACCCCCCC	0	0	35	17	99
113	GTAGCCCTACTAGC	0	0	36	18	74
114	GGATAGACTAACTG	0	0	35	18	49
115	GGGACTACTCATAA	0	0	43	19	52
116	GGTTAGCCTACTAG	0	0	35	18	71
117	GCCTAATAGGGGGG	0	0	38	20	64
118	GTACTACCCCTAGC	0	0	42	19	74
119	GCCTACTAGTAGCC	0	0	34	19	72
120	CCCTATAGGCCTAC	0	0	78	30	92
121	GTAGGATACCCCTA	0	0	36	19	73
122	GATAGGGGGTATAT	0	0	38	25	71
123	AGTACTACCCCCCC	0	0	38	21	104
124	TTACTAGTAGGGGC	0	0	34	17	54
125	GTACACTAGCTTAC	0	0	45	18	74
126	GTACCCTATATGAC	0	0	35	19	80
127	CTATACTATACCCC	0	0	36	16	75
128	GTAAGGGGGCCTAT	0	0	36	18	76
129	GGTTAGTTTAACCC	0	0	38	19	79
130	GTTTGATACCCTAC	0	0	51	22	82

Table S1 (Continued).

ID/Name	Sequence	Number of motifs		AST (Ul^{-1})	ALT (Ul^{-1})	T_m (˚C)
		TGC	TCC			
131	CAACCTAGTGGTAC	0	0	34	18	65
132	TGGACCCCTTACTA	0	0	37	19	59
133	GTTACCCCTATCTC	0	0	39	20	87
134	GGTGTAACTAGTTG	0	0	58	53	58
135	GTACAACTAGTGGG	0	0	36	20	56
136	GGGTAGCCTATTAG	0	0	41	22	62
137	GTACCCCTAGACTA	0	0	38	20	69
138	GCTAGTCAACTATC	0	0	50	36	64
139	CTATTGTACCCCTA	0	0	47	24	59
140	GGCCTACTAGTACC	0	0	33	16	81
141	GACTAGTCAACCCC	0	0	36	16	102
142	ACCTATTACCCCCC	0	0	49	25	109
143	GTTCAAGCTAGACC	0	0	41	14	91
144	TAGGTTAGTAGGGT	0	0	42	16	71
145	TATAGACCCCCCTA	0	0	43	16	72
146	GGGGCAACCTATAG	0	0	35	21	67
147	AGGATACCCCCCTA	0	0	44	17	78
148	TAGTTAGAGTATAC	0	0	38	19	63
149	CACTAGTATTAGGC	0	0	38	20	50

Table S2. TS-ASO sequences and their on-target mRNA transcripts. All oligonucleotides were fully modified with phosphorothioate linkages. Gap DNA and LNA are indicated in black and green font, respectively.

Name	Sequence	Number of motifs		Target Gene	Refseq ID
		TGC	TCC		
TS6-ASO	CTGTGATGACCTC	0	0	*Pcsk9*	NM_153565
TS7-ASO	ACACCAAGTTCTCC	0	1	*Pcsk9*	NM_153565
TS8-ASO	GTCTCTTTACCTGG	0	0	*Nr3c1*	NM_008173
TS9-ASO	AGGTGCTTTGGTCT	1	0	*Nr3c1*	NM_008173
TS10-ASO	CGCCCTCGCCCTC	0	0	*Rps6kb2*	NM_021485

Table S3. Primers used in the study for qRT-PCR analysis.

Gene		Primer Sequence
Gapdh	Forward	GTGTGAACGGATTTGGCCGT
	Reverse	GACAAGCTTCCCATTCTCGG
Pcsk9	Forward	GCTCAACTGTCAAGGGAAGG
	Reverse	CGTTGAGGATGCGGCTATAC
Nr3c1	Forward	ACTGTCCAGCATGCCGCTAT
	Reverse	GCAGTGGCTTGCTGAATTCC
Rps6kb2	Forward	GTGTGTCCCCTTGGCGAAT
	Reverse	CAGGTTCACGCTGCTCTCTGT

Table S4. Effect of nucleobase modification on the hepatotoxicity of TS-ASO.[a]

Nucleobase modification		ASO Name	Fold change vs control	
ID	Name		AST	ALT
-	Parent	TS1	361	1311
		TS2	45	139
		TS3	15	77
		TS6	25	40
		TS7	37	89.8
		TS8	94	337
		TS9	29	58
		TS10	8.2	26
C1	5-hydroxycytosine	TS1-C1-7	2.6	3.4
		TS1-C1-8	53	126
		TS1-C1-10	28	86
		TS1-C1-11	98	277
		TS2-C1-10	1.5	2.4
		TS3-C1-7	1.1	1.3
		TS6-C1-10	11	19
		TS7-C1-4	14	37
		TS7-C1-5	*	*
		TS7-C1-11	21	51
		TS8-C1-5	127	352
		TS8-C1-10	343	1309
		TS8-C1-11	60	146
		TS9-C1-6	32	43
		TS10-C1-3	7.9	33
		TS10-C1-4	7.2	28
		TS10-C1-5	7.1	25
		TS10-C1-7	4.5	8.7
		TS10-C1-9	2.1	5.3
		TS10-C1-10	3.2	9.1
C2	5-bromocytosine	TS1-C2-7	283	1153
C3	5-iodocytosine	TS1-C3-7	19	56
C4	5-(prop-1-yn-1-yl)cytosine	TS1-C4-7	178	650
		TS2-C4-10	6.5	30
		TS3-C4-7	4.7	25

< 5-fold	5~10-fold	10~20-fold	20~50-fold	>50-fold	*

Table S4 (Continued).

Nucleobase modification		ASO Name	Fold change vs control	
ID	Name		AST	ALT
T1	2-thio-thymine	TS1-T1-5	15	20
		TS2-T1-8	147	348
		TS3-T1-5	0.9	1.2
		TS6-T1-4	15	24
		TS6-T1-7	453	442
		TS7-T1-9	*	*
		TS7-T1-10	*	*
		TS8-T1-4	34	124
		TS8-T1-6	*	*
		TS8-T1-7	*	*
		TS8-T1-8	*	*
		TS9-T1-4	1.5	1.5
		TS9-T1-7	*	*
		TS9-T1-8	74	214
		TS9-T1-9	*	*
		TS10-T1-6	21	76
T2	5-fluorouracil	TS1-T2-5	150	425
		TS2-T2-8	25	88
		TS3-T2-5	2.0	5.5
T3	5-bromo-uracil	TS1-T3-5	436	1081
T5	5-hydroxymethyl-uracil	TS1-T5-5	47	132
G1	8-bromoguanine	TS1-G1-6	8.1	9.2
		TS2-G1-9	1.2	1.3
		TS3-G1-6	1.0	1.2
		TS6-G1-3	1.6	1.5
		TS6-G1-5	3.4	5.0
		TS6-G1-8	13	12
		TS7-G1-8	1.0	0.7
		TS9-G1-5	*	*
		TS9-G1-10	10	18
		TS9-G1-11	73	146
		TS10-G1-8	1.0	1.5

< 5-fold	5~10-fold	10~20-fold	20~50-fold	>50-fold	*

Table S4 (Continued).

Nucleobase modification		ASO Name	Fold change vs control	
ID	Name		AST	ALT
G2	8-aminoguanine	TS1-G2-6	5.6	6.5
		TS2-G2-9	1.3	1.7
		TS3-G2-6	1.3	2.3
		TS6-G2-3	2.1	2.4
		TS6-G2-5	10	11
		TS6-G2-8	3.4	2.9
		TS7-G2-8	1.0	0.7
		TS9-G2-5	8.1	17
		TS9-G2-10	23	38
		TS9-G2-11	75	100
		TS10-G2-8	1.0	1.1
G3	7-deazaguanine	TS1-G3-6	*	*
		TS2-G3-9	44	160
G4	7-iodo-<br>7-deazaguanine	TS1-G4-6	95	348
		TS2-G4-9	20	74
		TS7-G4-8	*	*
		TS9-G4-5	11	25
		TS9-G4-10	20	55
G5	7-(3-hydroxyprop-1-yn-1-yl)-<br>7-deazaguanine	TS1-G5-6	*	*
		TS2-G5-9	6.0	19
G6	7-phenylehtynyl-<br>7-deazaguanine	TS1-G6-6	30	37
		TS2-G6-9	1.5	3.5
		TS7-G6-8	2.9	3.7
		TS9-G6-5	51	86
		TS9-G6-10	42	107
		TS9-G6-11	71	119
G7	7-(pyridin-4-ylethynyl)-<br>7-deazaguanine	TS1-G7-6	10	16
		TS2-G7-9	1.6	2.5
		TS7-G7-8	1.7	1.0
		TS9-G7-5	20	34

< 5-fold	5~10-fold	10~20-fold	20~50-fold	>50-fold	*

[a]C57BL/6J mice were intravenously injected with TS-ASO or TS-ASO analogues with nucleobase modification at doses of 20 mg/kg. Ninety-six hours post injection, levels of serum AST, serum ALT were measured. *Mice were euthanised due to decreased spontaneous movement or died within 96 hours post administration.

Table S5. The index of overall changes for down-regulated genes classified up to *d*=2 and the extent of reduction of hepatotoxicity (AST and ALT values) of nucleobase modified gapmer ASOs.

	Index[a] (% of parent)	AST (% of parent)	ALT (% of parent)
TS1-C1-7	91.8	0.7	0.3
TS1-T1-5	87.1	4.1	1.5
TS1-G1-6	82.2	2.2	0.7
TS2-C1-10	75.5	3.2	1.7
TS3-C1-7	73.5	7.5	1.7

[a]The index was quantified by taking the logarithm of the ratio of gene expression changes and calculating the sum of the absolute value of it.

Table S6. The therapeutic index of TS6-ASO and TS6-ASO with nucleobase modification.[a]

	TD-1 (mg/kg)	TD-2 (mg/kg)	ED_{50} (mg/kg)	TI-1	TI-2
TS6	10	40	15.6	0.64	2.56
TS6-C1-10	20	＞80	25.7	0.78	＞3.1
TS6-G1-3	80	＞80	15.1	5.3	＞5.3
TS6-G2-3	80	＞80	14.7	5.4	＞5.4

[a]TI: The ratio of the toxic dose (TD) to the effective dose (ED50: the on-target gene expression was down-regulated to 50% of the level in the control group). TI-1: TD-1/ED50. TI-2: TD-2/ED50. TD-1: The dose that produces the >100 U/L elevation in the serum AST or ALT. TD-2: The dose concentration at which half the mice died.

Table S7. The T_m values of TS-ASOs or nucleobase-modified TS-ASOs used in this study.[a]

Name	Schematic illustration of TS-ASO	T_m (°C)	ΔT_m (°C)
	G T T A T G C C A C C C T A		
TS1	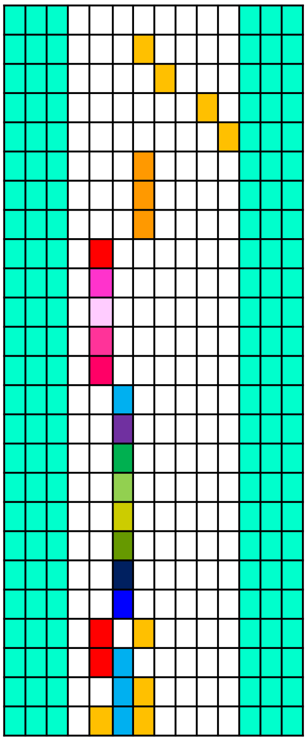	73	0
TS1-C1-7		71	-2
TS1-C1-8		61	-12
TS1-C1-10		62	-11
TS1-C1-11		61	-12
TS1-C2-7		74	1
TS1-C3-7		73	0
TS1-C4-7		75	2
TS1-T1-5		74	1
TS1-T2-5		71	-2
TS1-T3-5		74	1
TS1-T4-5		74	1
TS1-T5-5		73	0
TS1-G1-6		70	-3
TS1-G2-6		72	-1
TS1-G3-6		73	0
TS1-G4-6		74	1
TS1-G5-6		74	1
TS1-G6-6		73	0
TS1-G7-6		74	1
TS1-G8-6		73	0
TS1-C1T1		75	2
TS1-G1T1		72	-1
TS1-G1C1		69	-4
TS1-G1T1C1		71	-2
	G T C C G C A T G C C T A A		
TS2	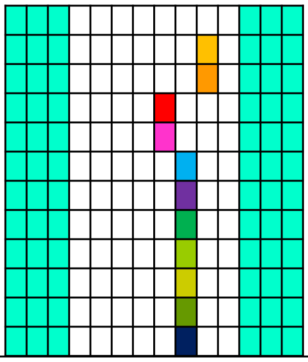	74	0
TS2-C1-10		73	-1
TS2-C4-10		76	2
TS2-T1-8		76	2
TS2-T2-8		73	-1
TS2-G1-9		71	-3
TS2-G2-9		72	-2
TS2-G3-9		74	0
TS2-G4-9		74	0
TS2-G5-9		75	1
TS2-G6-9		74	0
TS2-G7-9		75	1

Table S7 (Continued).

Name	Schematic illustration of TS-ASO	T_m (°C)	Δ T_m (°C)
	G A T A T G C C C T A C T A 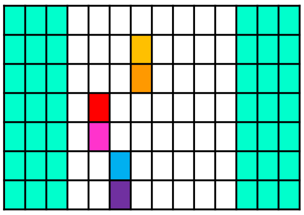		
TS3		66	0
TS3-C1-7		65	-1
TS3-C4-7		68	2
TS3-T1-5		67	1
TS3-T2-5		65	-1
TS3-G1-6		63	-3
TS3-G2-6		65	-1
	C T G T G A T G A C C T C 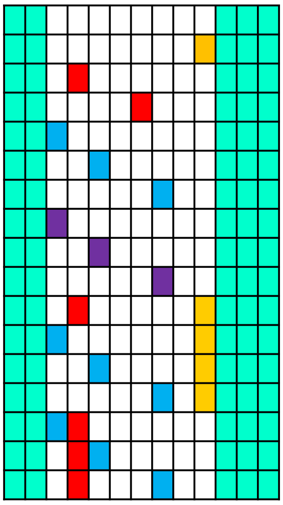		
TS6		63	0
TS6-C1-10		62	-1
TS6-T1-4		65	2
TS6-T1-7		65	2
TS6-G1-3		55	-8
TS6-G1-5		60	-3
TS6-G1-8		60	-3
TS6-G2-3		60	-3
TS6-G2-5		63	0
TS6-G2-8		62	-1
TS6-C1T1		63	0
TS6-G1C1-1		55	-8
TS6-G1C1-2		59	-4
TS6-G1C1-3		59	-4
TS6-G1T1-1		56	-7
TS6-G1T1-2		60	-3
TS6-G1T1-3		61	-2
	A C A C C A A G T T C T C C 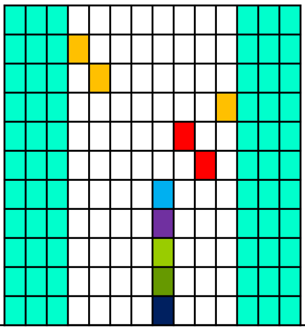		
TS7		66	0
TS7-C1-4		65	-1
TS7-C1-5		65	-1
TS7-C1-11		65	-1
TS7-T1-9		67	1
TS7-T1-10		66	0
TS7-G1-8		61	-5
TS7-G2-8		64	-2
TS7-G4-8		65	-1
TS7-G6-8		64	-2
TS7-G7-8		65	-1

Table S7 (Continued).

Name	Schematic illustration of TS-ASO	T_m (°C)	Δ T_m (°C)
	G T C T C T T T A C C T G G		
TS8	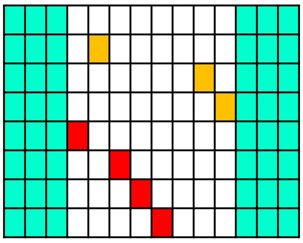	71	0
TS8-C1-5		71	0
TS8-C1-10		70	-1
TS8-C1-11		60	-11
TS8-T1-4		72	1
TS8-T1-6		71	0
TS8-T1-7		71	0
TS8-T1-8		72	1
	A G G T G C T T T G G T C T		
TS9	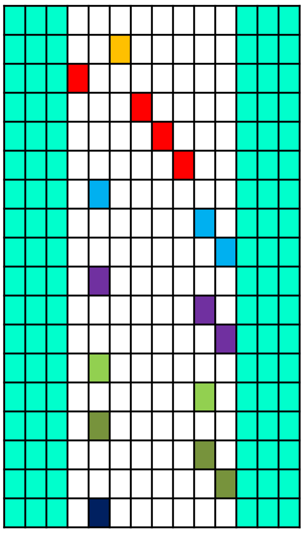	70	0
TS9-C1-6		69	-1
TS9-T1-4		71	1
TS9-T1-7		70	0
TS9-T1-8		70	0
TS9-T1-9		70	0
TS9-G1-5		66	-4
TS9-G1-10		66	-4
TS9-G1-11		64	-6
TS9-G2-5		69	-1
TS9-G2-10		68	-2
TS9-G2-11		68	-2
TS9-G4-5		71	1
TS9-G4-10		70	0
TS9-G6-5		70	0
TS9-G6-10		69	-1
TS9-G6-11		68	-2
TS9-G7-5		71	1
	C G C C C T C G C C C T C		
TS10	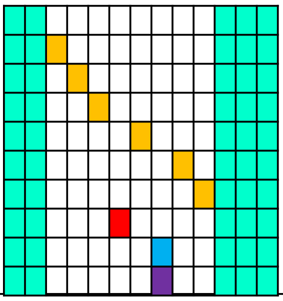	73	0
TS10-C1-3		72	-1
TS10-C1-4		73	0
TS10-C1-5		73	0
TS10-C1-7		73	0
TS10-C1-9		72	-1
TS10-C1-10		73	0
TS10-T1-6		74	1
TS10-G1-8		70	-3
TS10-G2-8		71	-2

[a]All samples contained 4 μM each strand, 10 mM sodium phosphate buffer (pH 7.2), and 100 mM NaCl. [b]The T_m value given is the average of the T_m values of three independent measurement. [c]ΔT_m values were calculated relative to the T_m values of the unmodified DNA/RNA duplexes.

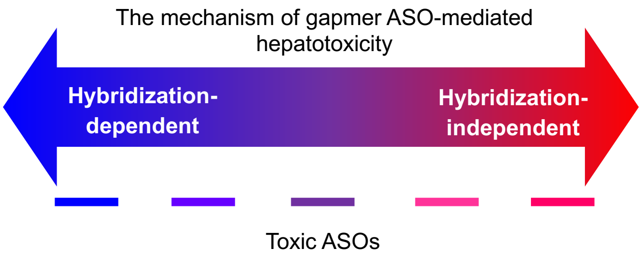

Figure S1. Schematic representation of screening of LNA gapmer ASOs. Mechanisms of gapmer ASO-mediated hepatotoxicity. Blue and red indicate hybridization-dependent and -independent mechanisms, respectively. Purple indicates a combination of hybridization-dependent and -independent mechanisms.

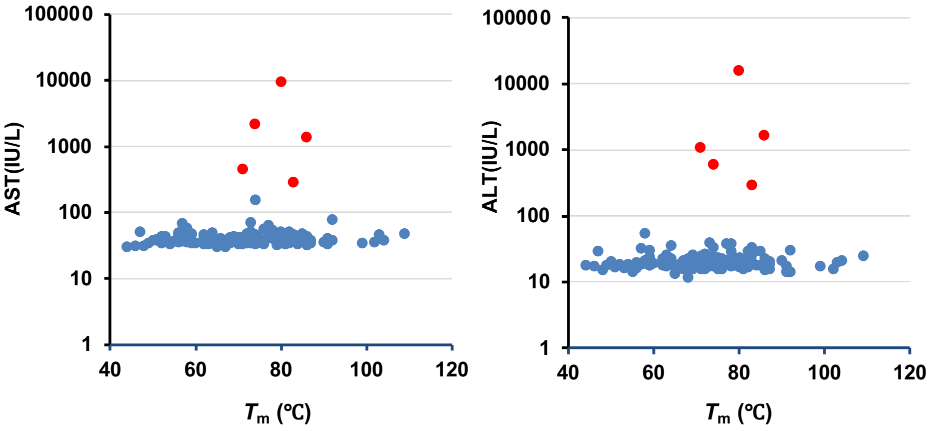

Toxic Ave 78.8(n=5)
Non-Toxic Ave 71.1(n=144)

Figure S2. Correlation between hepatotoxicity and Tm values of 149 LNA gapmers. Scatter plot. Logarithmic levels of serum AST (upper panels) and serum ALT (lower panels) are shown on the horizontal axis and predicted Tm values of the duplex formed between the LNA gapmer and RNA complement are shown on the vertical axis. The red dots indicate five TS-ASOs with serum AST and ALT levels over 100 U/L. The blue dots indicate the other 144 LNA gapmers with serum AST and ALT levels below 100 U/L.

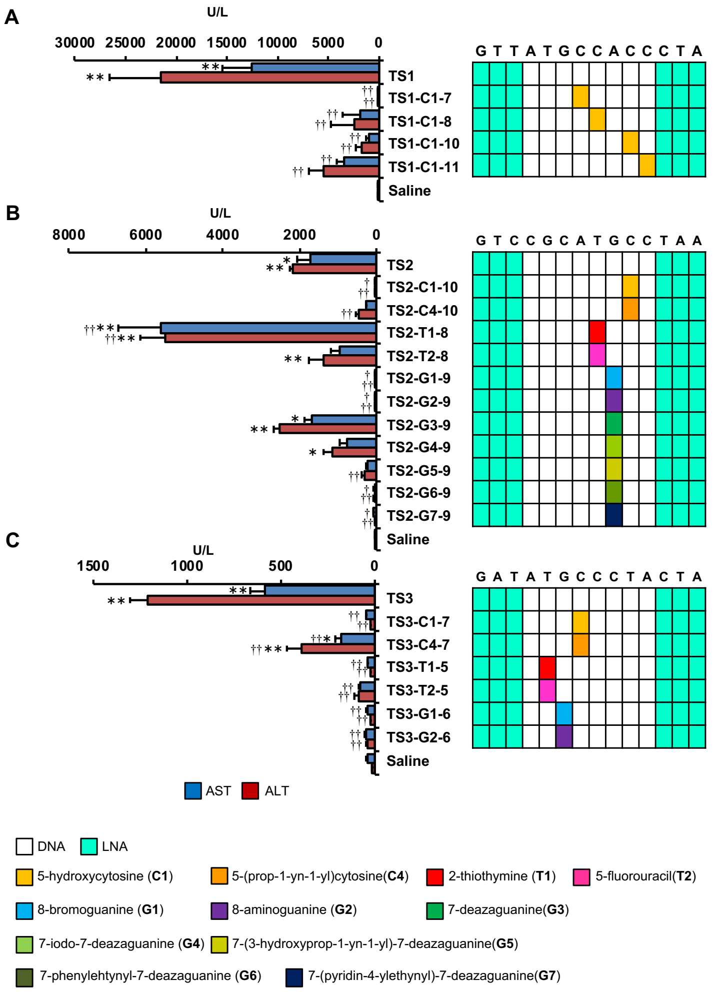

Figure S3. Effects of nucleobase modification on TS-ASOs hepatotoxicity and gene expression. Hepatotoxicity of TS1-ASO and TS1-ASO analogues with C1 (A), the TS2-ASO series (B), and the TS3-ASO series (C). Left panel: Serum AST and ALT levels. C57BL/6J mice were intravenously injected with TS-ASO or TS-ASO analogues with nucleobase modification at doses of 20 mg/kg. Ninety-six hours post injection, serum AST and ALT levels were measured. Results are expressed as mean ± S.E. (n = 4). Asterisks indicate a significant difference compared with that of the control group (**$p < 0.01$, *$p < 0.05$). Daggers indicate a significant difference compared with that of the parent TS-ASO-treated group (††$p < 0.01$, †$p < 0.05$). Right panel: Schematic of the parent TS-ASO and TS-ASO with nucleobase modifications in the gap region. (D) Scatter plots from microarray analysis of the cells treated with TS2-ASO, TS2-C1-10, TS3-ASO, and TS3-C1-7. The multiplied fluorescence intensity of control group is shown on the horizontal axis, and the proportion of change in gene expression as the results of introduction of each ASO (expressed logarithmically) is shown on the vertical axis. d: Distance; The total number of mismatches, insertions, or deletions between the ASO and complementary RNA sequences. Red dots: d=0 genes, which have perfect complementarity. Orange dots: d=1 genes. Light green dots: d=2 genes. Gray dots: $d \geqq 3$ genes. (E) The relationship between hepatotoxicity (AST and ALT values) and the index of overall down-regulation. The levels of serum AST and ALT in mice treated with TS2-ASO or TS2-C1-10, TS3-ASO or TS3-C1-7 are shown on the vertical axis. The index of overall changes for down-regulated genes classified up to d = 2 in the cells treated with TS2-ASO or TS2-C1-10, TS3-ASO or TS3-C1-7 is shown on the horizontal axis. The index was quantified by taking the logarithm of the ratio of gene expression changes and calculating the sum of the absolute value of it (see a conceptual diagram of the index in Fig. S4). Blue plot indicates parent ASO, and each color indicates each TS-ASO analogues with nucleobase modification.

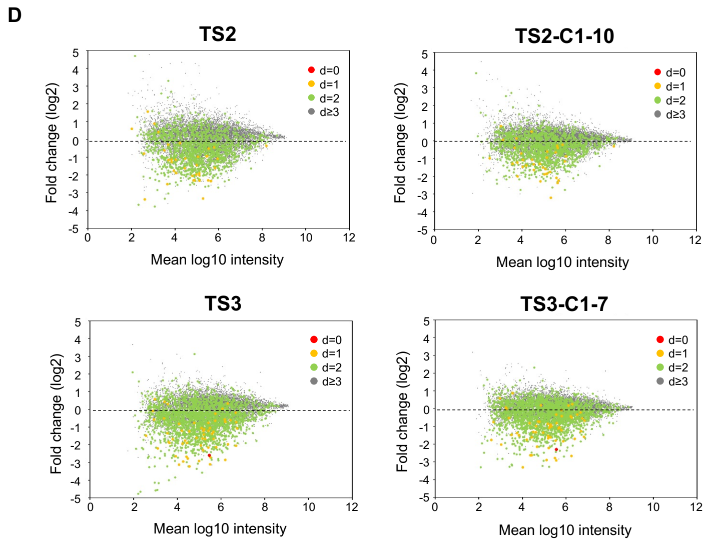

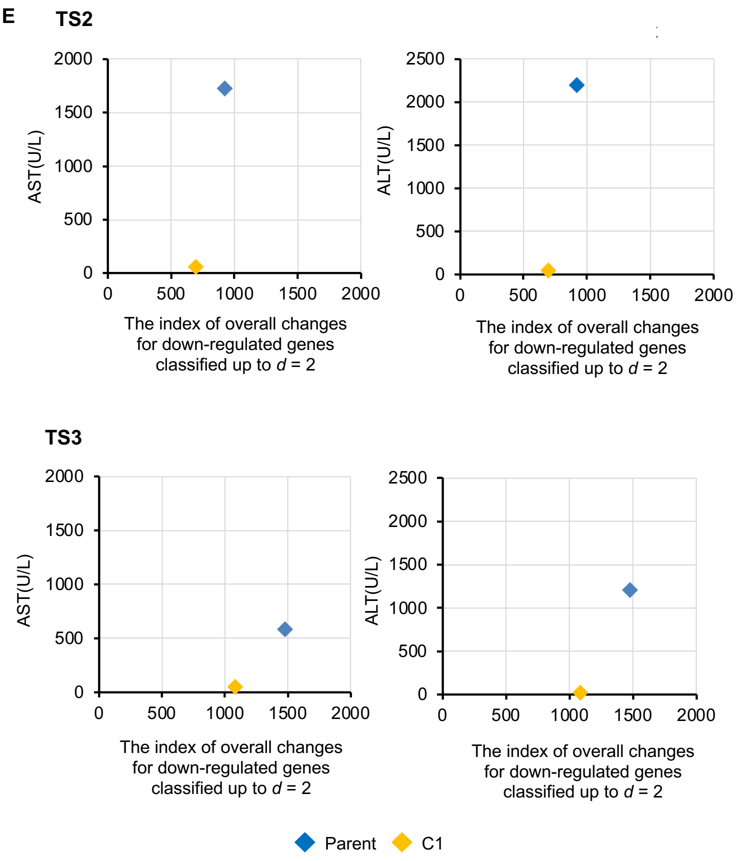

Figure S3 (Continued).

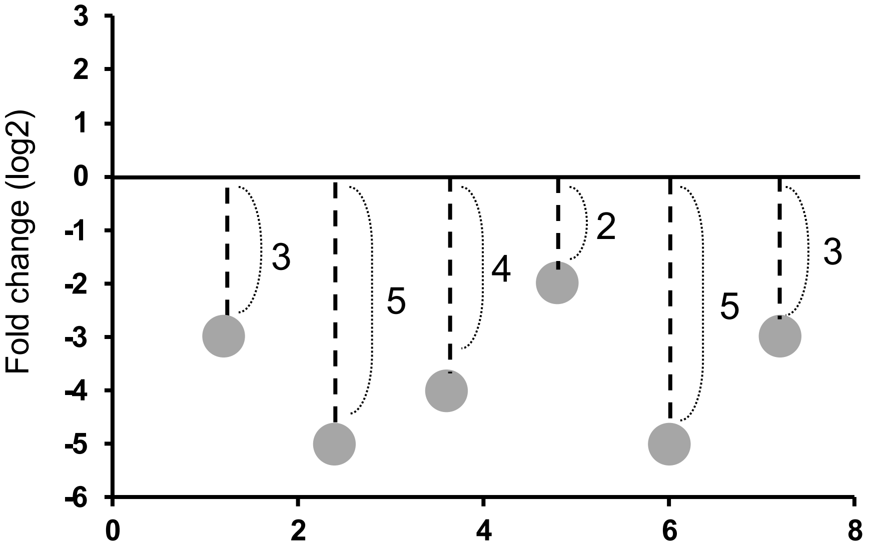

Index of overall changes for down-regulated genes:

The sum of the absolute values of the changes (the logarithm of the ratio) in gene expression of down-regulated genes

= 3 + 5 + 4 + 2 + 5 +3

= **21**

Figure S4. The concept of the quantitative analysis method of the overall changes in gene expression. Exemplified scatter plot and the calculation method of index of overall changes for down-regulated genes are shown.

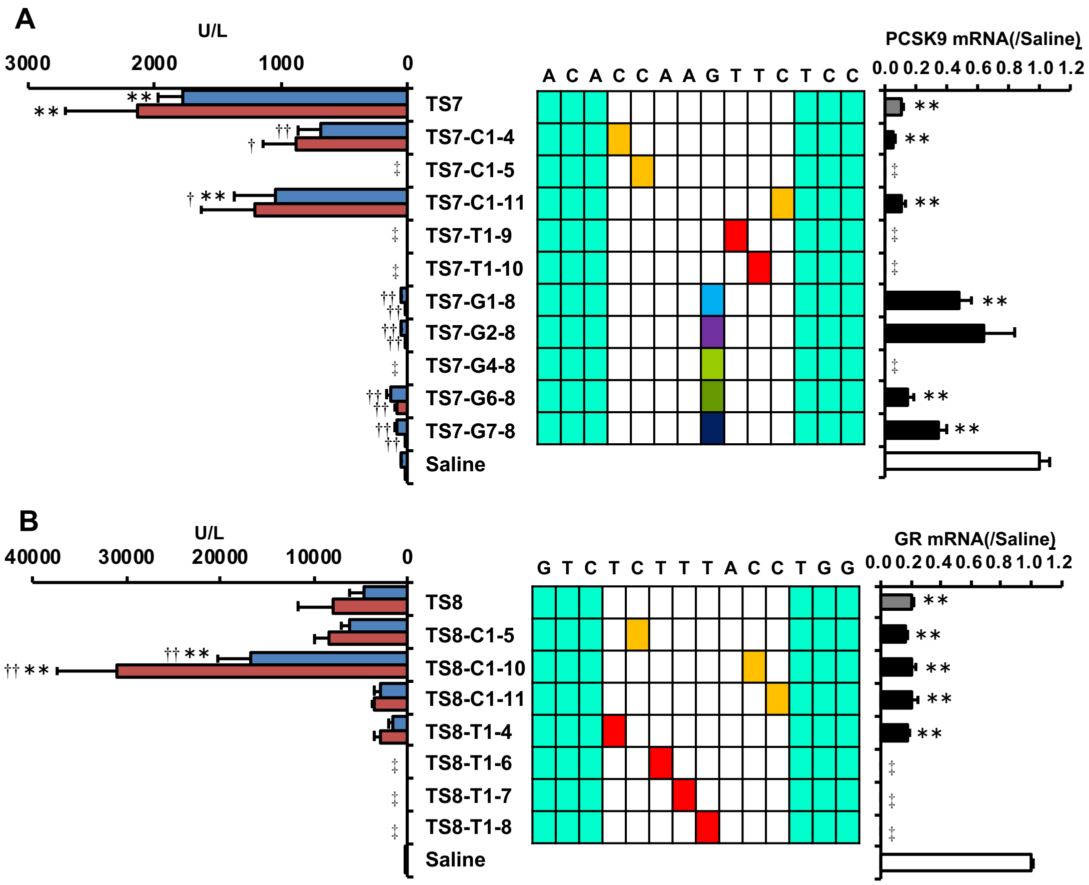

Figure S5. Effect of nucleobase modification on TS-ASO hepatotoxicity and on-target activity. Hepatotoxicity of the TS7-ASO series (A), TS8-ASO series (B), TS9-ASO series (C), and TS10-ASO series (D). Left panel: Serum AST and ALT levels. Right panel: On-target activity. C57BL/6J mice were intravenously injected with TS-ASO or TS-ASO analogues with nucleobase modification at doses of 20 mg/kg. Ninety-six hours post injection, levels of serum AST, serum ALT, and Pcsk9 mRNA (TS7-ASO target) (A), GR mRNA (TS8-ASO and TS9-ASO target) (B, C), and Rbs6kb2 mRNA (TS10-ASO target) (D) were measured. ‡Mice were euthanised due to decreased spontaneous movement or dead within 96 hours post administration. Results are expressed as mean ± S.E. (n = 4). Asterisks indicate a significant difference compared with that of the control group (***p* < 0.01, **p* < 0.05). Daggers indicate a significant difference compared with that of the parent TS-ASO-treated group (††*p* < 0.01, †*p* < 0.05). Middle panel: Schematic of the parent TS-ASO and TS-ASO analogues with nucleobase modifications in the gap region.

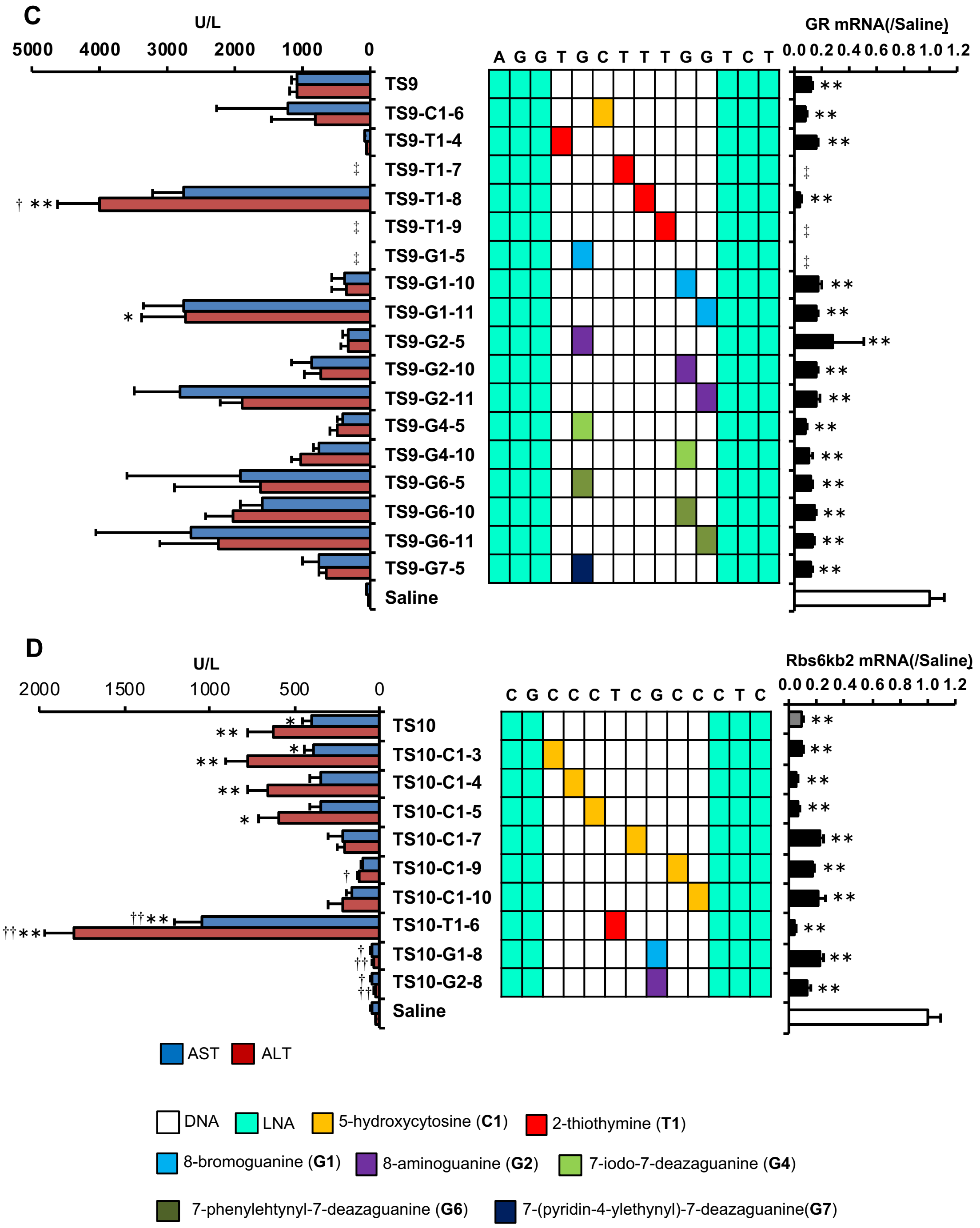

Figure S5 (Continued).

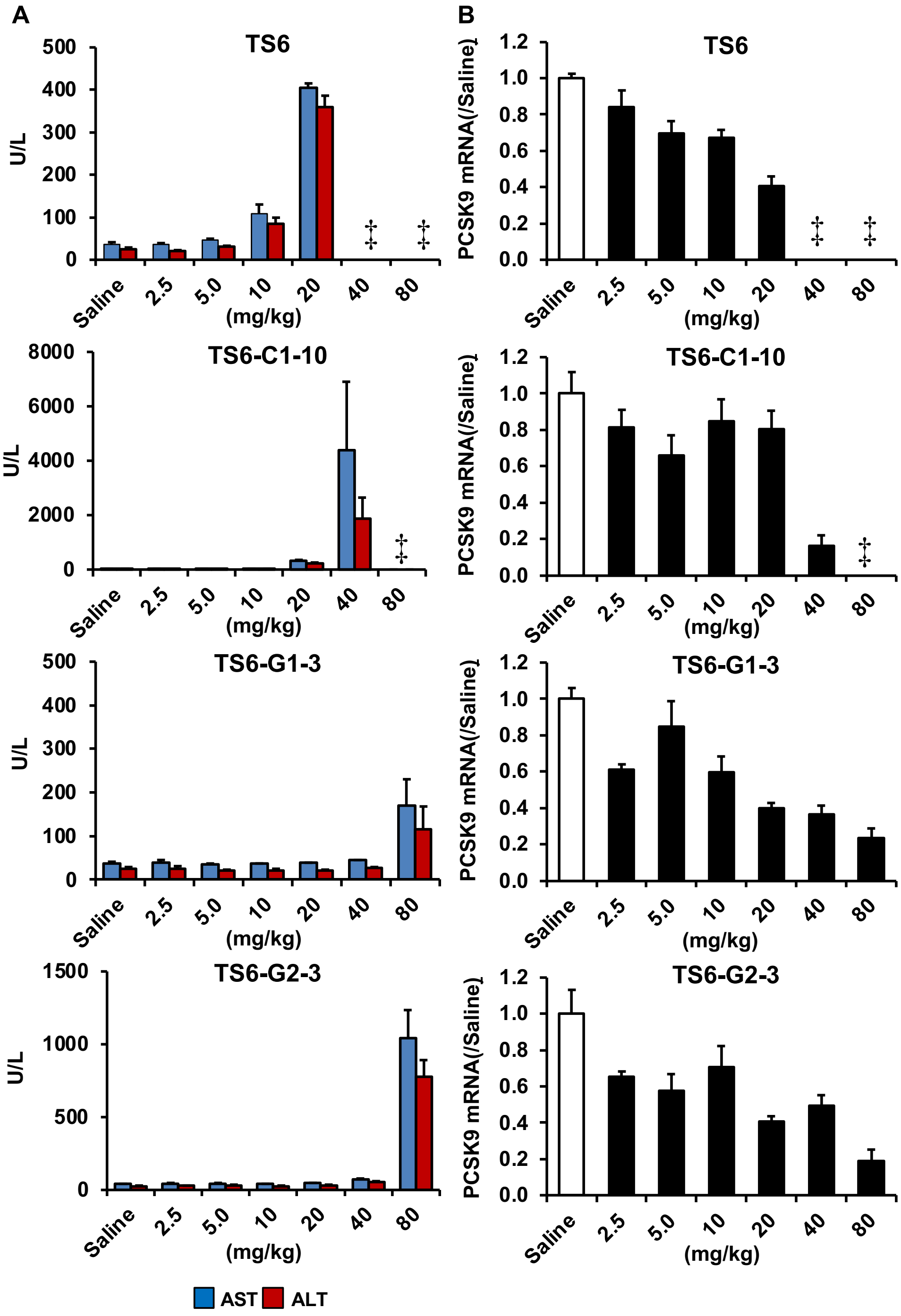

Figure S6. The dose-responses of effect of nucleobase modification on TS6-ASO hepatotoxicity and on-target activity. Hepatotoxicity (A) and on-target activity (B) of TS6-ASO series. C57BL/6J mice were intravenously injected with TS6-ASO or TS6-ASO analogues with nucleobase modification at doses of 2.5-80 mg/kg. Ninety-six hours post injection, levels of serum AST, serum ALT, and *Pcsk9* mRNA (TS6-ASO target) were measured. ‡Mice were euthanised due to decreased spontaneous movement or died within 96 hours post administration. Results are expressed as mean ± S.E. (n = 4).

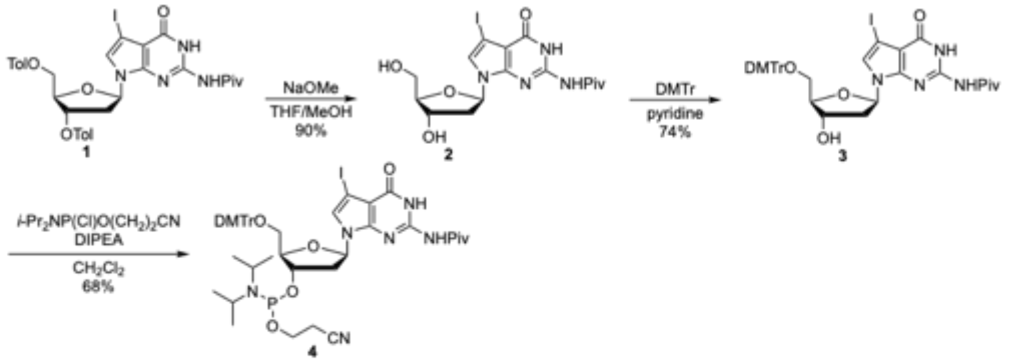

Scheme S1. Synthesis of G4 phosphordite.

Synthesis of 2. To a solution of compound **1**[1] (4.5 g, 6.2 mmol) in THF/MeOH (THF/MeOH=100/1, 151.5 mL) were added 1 M NaOMe (MeOH solution, 12.4 mL, 12.4 mmol) at 0˚C, and the reaction mixture was stirred at 0˚C for 15 min. To the reaction mixture AcOH (0.8 mL) were added at 0˚C and the solvent was evaporated to dryness. The crude mixture was purified by silica gel column chromatography ($CHCl_3$/MeOH = 9/1). Compound **2** was isolated in 90% yield (3.0 g). ^{1}H NMR (500 MHz, DMSO-d_6) δ: 1.25 (9H, s), 2.12.13 (1H, m), 2.35-2.40 (1H, m), 3.47-3.55 (2H, m), 3.78 (1H, m), 4.31 (1H, m), 4.96 (1H, t, *J* = 5.7 Hz), 5.26 (1H, d, *J* = 3.1 Hz), 6.46 (1H, dd, *J* = 5.0 and 5.5 Hz), 7.46 (1H, s), 10.98 (1H, s), 11.93 (1H, s). ^{13}C NMR (101 MHz, DMSO-d_6) δ: 181.18, 156.26, 147.69, 147.45, 124.41, 103.99, 87.32, 82.46, 70.96, 61.81, 55.85, 40.15, 40.02, 39.63, 26.33. HRMS calc. for $C_{16}H_{21}IN_4O_5Na$ $[M+Na]^+$: 499.0449; found: 499.0447.

Synthesis of 3. To a solution of compound **2** (0.8 g, 2.4 mmol) in pyridine (15 mL) were added 4,4-dimethoxytritylchloride (0.8 g, 2.4 mmol), and the mixture was stirred at room temperature overnight. To the reaction mixture MeOH (5 mL) was added, and the mixture was stirred for 1 h. The reaction mixture was diluted with $CHCl_3$ and washed by saturated $NaHCO_3$ (aq.) and brine, and dried over Na_2SO_4. The organic phase was evaporated to dryness and purified by silica gel column chromatography ($CHCl_3$/MeOH/NEt_3 = 94/5/1). Compound **3** was isolated in 74% yield (1.3 g). ^{1}H NMR (300 MHz, $CDCl_3$) δ: 1.30 (9H, s), 1.98 (1H, d, *J* = 3.4 Hz), 2.36 (1H, m), 2.44-2.49 (1H, m), 3.28 (1H, dd, *J* = 6.0 and 3.0 Hz), 3.40 (1H, dd, *J* = 6.2 and 2.6 Hz), 3.80 (6H, m), 4.04 (1H, m), 4.55 (1H, m), 6.42 (1H, t, *J* = 6.7 Hz), 6.83-6.85 (4H, m), 6.99 (1H, s), 7.22-7.25 (1H, m), 7.29-7.33 (6H, m), 7.41-7.43 (2H, m), 7.93 (1H, s), 11.69 (1H, s). ^{13}C NMR (126 MHz, $CDCl_3$) δ: 180.12, 158.65, 157.19, 147.59, 146.61, 144.52, 135.74, 135.59, 130.18, 130.12, 128.22, 128.09, 127.09, 124.19, 113.36, 105.53, 86.76, 85.95, 83.17, 72.71, 64.09, 55.60, 55.37, 40.53, 40.27, 26.99. HRMS calc. for $C_{37}H_{39}IN_4O_7Na$ $[M+Na]^+$: 801.1756; found: 801.1758.

Synthesis of 4. To a solution of compound **3** (0.5 g, 0.6 mmol) in CH_2Cl_2 (30 mL) were added *N*,*N*-diisopropylethylamine (0.3 mL, 1.7 mmol) and *i*-$Pr_2NP(Cl)O(CH_2)_2CN$ (0.3 mL, 1.2 mmol),

and the mixture was stirred at room temperature for 1 h. The reaction mixture was washed by saturated $NaHCO_3$ (aq.) and brine, dried over Na_2SO_4. The organic phase was evaporated to dryness and purified by silica gel column chromatography (Hexane/AcOEt/NEt_3 = 10/9/1). Compound **4** was isolated in 68% yield (0.4 g). ^{31}P NMR (122 MHz, $CDCl_3$) δ: 148.32, 147.94. ^{1}H NMR (300 MHz, $CDCl_3$) δ: 1.11-1.21 (13H, m), 1.28 (9H, s), 2.42-2.57 (2H, m), 2.67 (1H, m), 3.23-3.34 (2H, m), 3.53-3.75 (3H, m), 3.79 (6H, s), 3.82-3.89 (1H, m), 4.23 (1H, m), 4.59-4.68 (1H, m), 5.29 (2H, s), 6.39-6.45 (1H, m), 6.83 (4H, m), 7.05 (1H, m), 7.20-7.34 (7H, m), 7.42-7.44 (2H, m), 8.15 (0.5H, s), 8.36 (0.5H, s), 11.77 (1H, s). ^{13}C NMR (126 MHz, $CDCl_3$) δ: 180.07, 179.78, 158.68, 157.05, 156.96, 147.47, 147.39, 146.65, 146.52, 144.56, 135.80, 135.75, 135.65, 135.59, 130.26, 130.18, 130.12, 128.30, 128.22, 128.07, 127.07, 124.16, 124.12, 117.62, 113.34, 105.83, 105.77, 86.71, 86.68, 85.50, 85.46, 85.40, 83.84, 83.48, 77.37, 74.60, 74.41, 73.90, 73.74, 63.84, 63.74, 58.61, 58.42, 58.39, 58.21, 55.65, 55.39, 43.39, 43.35, 43.26, 43.23, 40.33, 40.28, 40.18, 40.14, 39.80, 39.77, 27.07, 27.02, 24.73, 24.65, 20.54, 20.49, 20.43, 20.36. HRMS calc. for $C_{46}H_{56}IN_6O_8PNa$ $[M+Na]^+$: 1001.2834; found: 1001.2833.

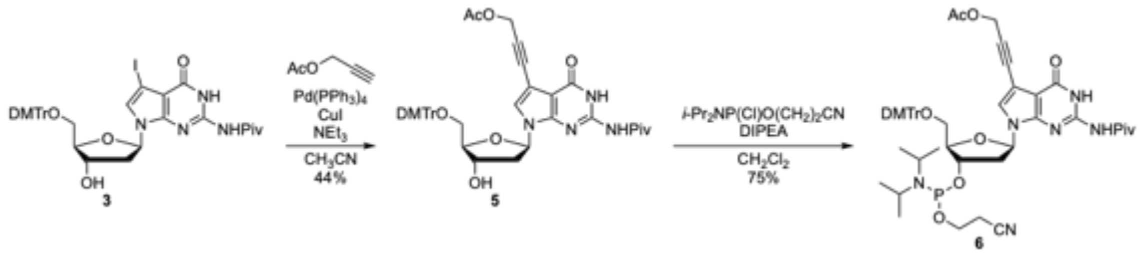

Scheme S2. Synthesis of G5 phosphoramidite.

Synthesis of 5. To a solution of compound **3** (0.1 g, 0.1 mmol) in MeCN (2.5 mL) were added CuI (4.0 mg, 0.01 mmol), NEt_3 (0.09 mL, 0.7 mmol), 2-propynyl acetate (0.02 g, 0.2 mmol) and tetrakis(triphenylphosphine)palladium(0) (0.02 mg, 0.01 mmol), and the mixture was stirred at 80˚C for 2 h. The reaction mixture was filtrated through Celite pad and evaporated to dryness. The crude mixture was purified by silica gel column chromatography ($CHCl_3$/MeOH/NEt_3 = 98/1/1) and amino silica gel chromatography ($CHCl_3$/MeOH/NEt_3 = 98/1/1). Compound **5** was isolated in 44% yield (0.08 g). ^{1}H NMR (300 MHz, $CDCl_3$) δ: 1.30 (9H, s), 2.10 (3H, s), 2.17 (1H, d, *J* = 3.2 Hz), 2.36-2.40 (2H, m), 3.26 (1H, dd, *J* = 10.1 and 5.6 Hz), 3.39 (1H, dd, *J* = 9.8 and 4.4 Hz), 3.79 (6H, s), 4.03 (1H, dd, *J* = 8.3 and 5.0 Hz), 4.52 (1H, m), 4.92 (2H, s), 6.40 (1H, t, *J* = 6.7 Hz), 6.82-6.85 (4H, m), 7.08 (1H, s), 7.20-7.25 (1H, m), 7.29-7.32 (6H, m), 7.40-7.43 (2H, m), 8.04 (1H, s), 11.74 (1H, s). ^{13}C NMR (101 MHz, $CDCl_3$) δ: 179.99, 170.74, 158.68, 156.88, 147.25, 147.14, 144.64, 135.74, 135.67, 130.13, 128.22, 128.07, 127.07, 124.16, 113.34, 105.07, 100.09, 86.70, 85.75, 84.74, 83.00, 79.36, 72.60, 64.07, 55.34, 53.59, 40.30, 40.20, 27.05, 20.97. HRMS calc. for $C_{42}H_{44}N_4O_9Na$ $[M+Na]^+$: 771.3001; found: 771.3002.

Synthesis of 6. To a solution of compound **5** (0.3 g, 0.4 mmol) in CH_2Cl_2 (20 mL) were added *N*,*N*-diisopropylethylamine (0.2 mL, 1.0 mmol) and *i*-$Pr_2NP(Cl)O(CH_2)_2CN$ (0.1 mL, 0.4 mmol), and the mixture was stirred at room temperature for 1 h. The reaction mixture was washed by saturated $NaHCO_3$ (aq.) and brine, dried over Na_2SO_4. The organic phase was evaporated to dryness and purified by silica gel column chromatography (Hexane/AcOEt/NEt_3 = 10/9/1). Compound **6** was isolated in 75% yield (0.3 g). ^{1}H NMR (300 MHz, $CDCl_3$) δ: 1.11-1.21 (13H, m), 1.29 (10H, s), 2.09 (3H, s), 2.37-2.46 (1H, m), 2.51 (1H, m), 2.56-2.61 (0.5H, m), 2.67 (1H, m), 3.20-3.33 (2H, m), 3.53-3.77 (3H, m), 3.79 (6H, s), 3.81-3.91 (1H, m), 4.20-4.26 (1H, m), 4.57-4.66 (1H, m), 4.92 (2H, s), 6.37-6.43 (1H, m), 6.82 (4H, m), 7.08-7.14 (1H, m), 7.21-7.24 (1H, m), 7.28-7.34 (6H, m), 7.42 (2H, m), 8.09 (0.5H, s), 8.32 (0.5H, s), 11.78 (1H, s). ^{13}C NMR (101 MHz, $CDCl_3$) δ: 180.07, 179.78, 170.56, 158.67, 156.82, 156.74, 147.18, 147.12, 147.05, 144.66, 144.63, 135.75, 135.72, 135.69, 135.64, 130.24, 130.17, 130.14, 128.28, 128.21, 128.04, 127.04, 124.26, 117.62, 113.31, 105.31, 105.27, 100.14, 99.87, 86.63, 85.42, 84.68, 84.52, 83.86, 83.46, 79.47, 79.31, 77.36, 74.47, 74.28, 73.69, 73.53, 63.74, 63.65, 58.62, 58.44, 58.33, 58.15, 55.34,

53.56, 53.52, 43.41, 43.36, 43.28, 43.24, 40.33, 40.28, 39.98, 29.81, 27.07, 27.02, 24.74, 24.67, 20.95, 20.56, 20.50, 20.42, 20.35. ^{31}P NMR (122 MHz, $CDCl_3$) δ: 148.13, 148.45. HRMS calc. for $C_{51}H_{61}N_6O_{10}PNa$ $[M+Na]^+$: 971.4079; found: 971.4062.

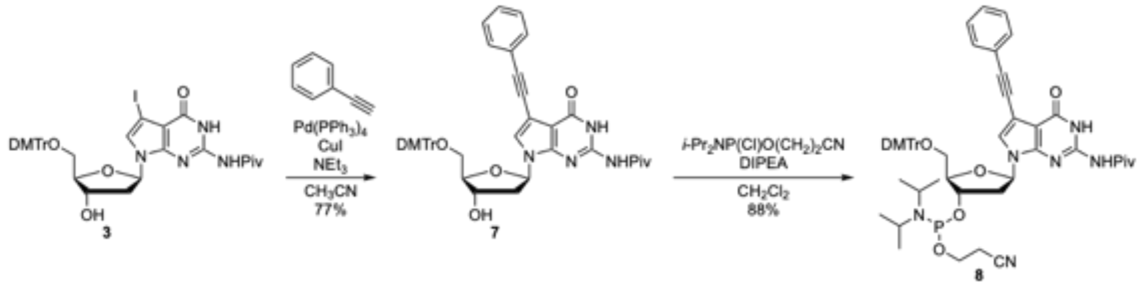

Scheme S3. Synthesis of G6 phosphoramidite.

Synthesis of 7. To a solution of compound **3** (0.2 g, 0.3 mmol) in MeCN (5 mL) were added CuI (6.0 mg, 0.03 mmol), NEt_3 (0.09 mL, 0.6 mmol), ethynylbenzene (0.04 mL, 0.4 mmol) and tetrakis(triphenylphosphine)palladium(0) (0.035 mg, 0.03 mmol), and the mixture was stirred at 80˚C for 2 h. The reaction mixture was filtrated through Celite pad and evaporated to dryness. The crude mixture was purified by silica gel column chromatography (AcOEt/NEt_3 = 98/2). Compound **7** was isolated in 77% yield (0.2 g). ^{1}H NMR (300MHz, $CDCl_3$) δ: 1.32 (9H, s), 1.92 (1H, d, *J* = 3.7 Hz), 2.40-2.48 (2H, m), 3.29 (1H, dd, *J* = 9.9 and 5.1 Hz), 3.42 (1H, dd, *J* = 10.2 and 4.5 Hz), 3.74 (6H, s), 4.05 (1H, q, *J* = 3.9 Hz), 4.55 (1H, m), 6.45 (1H, t, *J* = 6.7 Hz), 6.84 (4H, m), 7.14 (1H, s), 7.19-7.24 (1H, m), 7.28-7.34 (9H, m), 7.43 (2H, m), 7.53 (2H, m), 7.92 (1H, s), 11.70 (1H, s). ^{13}C NMR (101MHz, $CDCl_3$) δ: 179.93, 158.69, 156.98, 147.32, 147.08, 144.61, 135.76, 135.67, 131.70, 130.16, 128.22, 128.08, 127.95, 127.10, 123.81, 123.12, 113.35, 105.05, 101.40, 91.25, 86.74, 85.77, 83.02, 82.24, 72.66, 64.08, 55.31, 40.29, 27.07. HRMS calc. for $C_{45}H_{44}N_4O_7Na$ $[M+Na]^+$: 775.3102; found: 775.3100.

Synthesis of 8. To a solution of compound **7** (0.4 g, 0.5 mmol) in CH_2Cl_2 (20 mL) were added *N*,*N*-diisopropylethylamine (0.2 mL, 1.2 mmol) and *i*-$Pr_2NP(Cl)O(CH_2)_2CN$ (0.2 mL, 0.7 mmol), and the mixture was stirred at room temperature for 1 h. The reaction mixture was washed by saturated $NaHCO_3$ (aq.) and brine, dried over Na_2SO_4. The organic phase was evaporated to dryness and purified by silica gel column chromatography (Hexane/AcOEt/NEt_3 = 10/9/1). Compound **8** was isolated in 88% yield (0.4 g). ^{31}P NMR (162MHz, $CDCl_3$) δ: 148.27, 147.98. ^{1}H NMR (400 MHz, $CDCl_3$) δ: 1.13 (3H, d, J=6.7 Hz), 1.17-1.21 (11H, m), 1.30 (11H, s), 1.33 (1H, m), 1.66 (2H, s), 2.41-2.55 (3H, m), 2.58-2.74 (1.5H, m), 3.23-3.35 (2H, m), 3.53-3.67 (2H, m), 3.73 (8H, s), 3.76-3.89 (1.5H, m), 4.22-4.28 (1H, m), 4.60-4.68 (1H, m), 6.45 (1H, m), 6.75-6.84 (5.5H, m), 7.18-7.22 (3H, m), 7.27-7.35 (11H, m), 7.43-7.45 (2H, m), 7.50-7.53 (2H, m), 8.06 (0.5H, s), 8.29 (0.5H, s), 11.77 (1H, m). ^{13}C NMR (126 MHz, $CDCl_3$) δ: 180.02, 179.75, 158.66, 156.93, 156.84, 147.21, 147.15, 147.03, 144.62, 135.78, 135.75, 135.71, 135.65, 131.74, 130.22, 130.15, 128.32, 128.24, 128.18, 128.05, 127.88, 127.83, 127.05, 123.94, 123.86, 123.22, 123.15, 117.64, 113.31, 105.28, 105.24, 101.43, 101.17, 91.26, 91.11, 86.64, 86.60, 85.42, 85.37, 83.84, 83.46, 82.36, 82.20, 74.46, 74.32, 73.72, 73.58, 63.83, 63.70, 58.61, 58.46, 58.35, 58.21, 55.31,

43.39, 43.35, 43.30, 43.24, 40.33, 40.29, 40.06, 40.03, 39.68, 39.66, 29.83, 27.11, 27.06, 24.74, 24.68, 20.56, 20.51, 20.43, 20.37. HRMS calc. for $C_{54}H_{61}N_6O_8PNa$ $[M+Na]^+$: 975.4181; found: 975.4181.

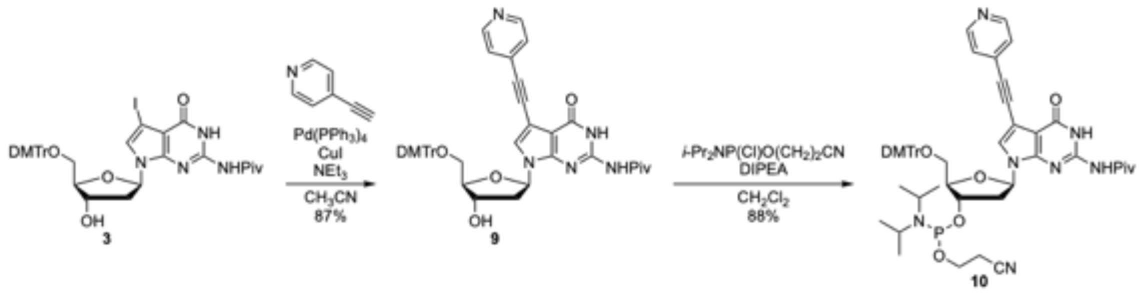

Scheme S4. Synthesis of G7 phosphoramidite.

Synthesis of 9. To a solution of compound **3** (0.1 g, 0.1 mmol) in MeCN (2.5 mL) were added CuI (2.0 mg, 0.01 mmol), NEt_3 (0.04 mL, 0.3 mmol), *p*-ethynylpyridine (0.02 g, 0.2 mmol) and tetrakis(triphenylphosphine)palladium(0) (0.01 mg, 0.01 mmol), and the mixture was stirred at 80˚C for 2 h. The reaction mixture was filtrated through Celite pad and evaporated to dryness. The crude mixture was purified by silica gel column chromatography (AcOEt/NEt_3 = 98/2). Compound **9** was isolated in 87% yield (0.08 g). ^{1}H NMR (300 MHz, $CDCl_3$) δ: 1.33 (9H, s), 2.22 (1H, d, *J* = 2.8 Hz), 2.41-2.49 (2H, m), 3.31 (1H, dd, *J* = 10.2 and 4.5 Hz), 3.41 (1H, dd, *J* = 10.1 and 4.7 Hz), 3.75 (6H, s), 4.08 (1H, m), 4.58 (1H, s), 6.42 (1H, t, *J* = 6.7 Hz), 6.82-6.85 (4H, m), 7.18-7.24 (3H, m), 7.30-7.44 (9H, m), 8.15 (1H, s), 8.55 (2H, m), 11.79 (1H, s). ^{13}C NMR (126 MHz, $CDCl_3$) δ: 180.38, 158.66, 157.08, 149.37, 147.71, 147.51, 144.66, 135.84, 135.72, 132.44, 130.19, 130.16, 128.28, 128.08, 127.06, 125.83, 124.54, 113.33, 104.93, 100.03, 88.70, 87.78, 86.71, 86.34, 83.33, 72.50, 64.17, 55.31, 40.53, 40.43, 27.07. HRMS calc. for $C_{44}H_{43}N_5O_7Na$ $[M+Na]^+$: 776.3055; found: 776.3054.

Synthesis of 10. To a solution of compound **9** (0.5 g, 0.7 mmol) in CH_2Cl_2 (30 mL) were added *N*,*N*-diisopropylethylamine (0.3 mL, 1.7 mmol) and *i*-$Pr_2NP(Cl)O(CH_2)_2CN$ (0.2 mL, 0.7 mmol), and the mixture was stirred at room temperature for 1 h. The reaction mixture was washed by saturated $NaHCO_3$ (aq.) and brine, dried over Na_2SO_4. The organic phase was evaporated to dryness and purified by silica gel column chromatography (Hexane/AcOEt/NEt_3 = 10/9/1). Compound **10** was isolated in 88% yield (0.4 g). ^{31}P NMR (122 MHz, $CDCl_3$) δ: 148.48, 148.05. ^{1}H NMR (300 MHz, $CDCl_3$) δ: 1.12-1.21 (16H, m), 1.30 (9H, s), 2.45-2.54 (3H, m), 2.60-2.71 (2H, m), 3.26-3.35 (2H, m), 3.54-3.70 (3H, m), 3.74 (6H, s), 3.82-3.90 (2H, m), 4.26 (1H, m), 4.67 (1H, m), 6.44 (1H, m), 6.82 (4H, m), 7.20-7.23 (2H, m), 7.28-7.37 (11H, m), 7.45 (3H, m), 8.19 (0.5H, s), 8.42 (0.5H, s), 8.54 (2H, m), 11.84 (1H, m). ^{13}C NMR (101 MHz, $CDCl_3$) δ: 180.18, 179.91, 158.64, 156.82, 156.74, 149.51, 147.38, 147.28, 144.56, 135.71, 135.68, 135.65, 135.60, 132.20, 132.13, 130.18, 130.12, 128.66, 128.53, 128.28, 128.19, 128.01, 127.04, 125.59, 124.56, 124.47, 117.64, 117.62, 113.28, 105.14, 100.15, 99.84, 88.63, 88.52, 87.75, 87.53, 86.66, 86.63, 85.55, 85.49, 84.13, 83.67, 77.37, 74.39, 74.21, 73.58, 73.42, 63.70, 63.58, 58.55, 58.37, 58.25, 58.07, 55.26, 43.36, 43.32, 43.25, 43.20, 40.33, 40.29, 39.96, 29.76, 28.84, 27.03, 26.98, 24.70, 24.62,

20.54, 20.49, 20.39, 20.32, 19.52. HRMS calc. for $C_{53}H_{60}N_7O_8PNa$ $[M+Na]^+$: 976.4133; found: 976.4121.

NMR spectra of compound 2

^{1}H NMR

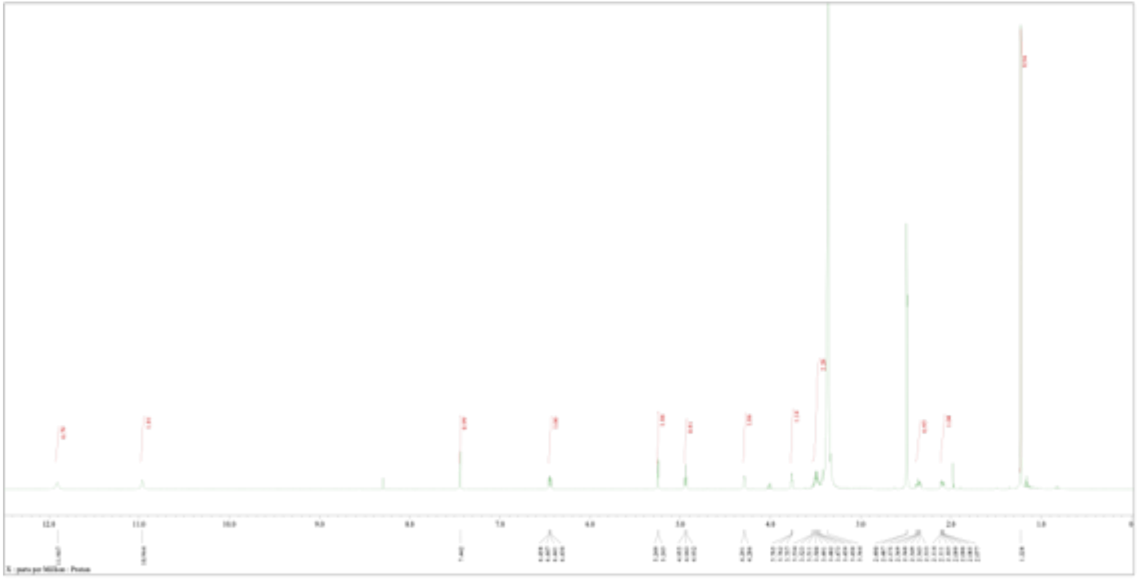

^{13}C NMR

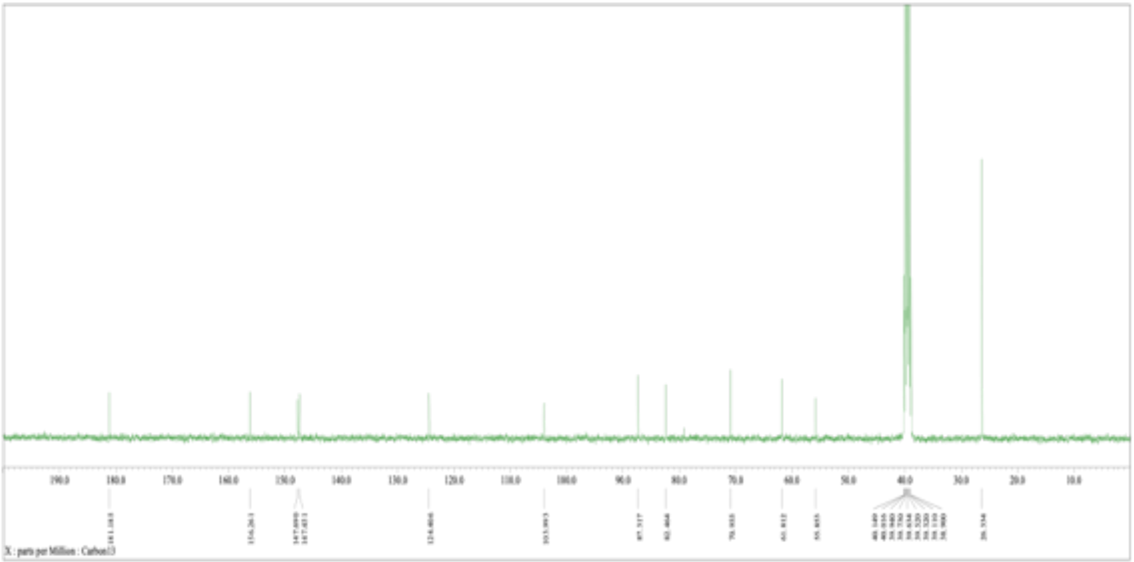

NMR spectra of compound 3

^{1}H NMR

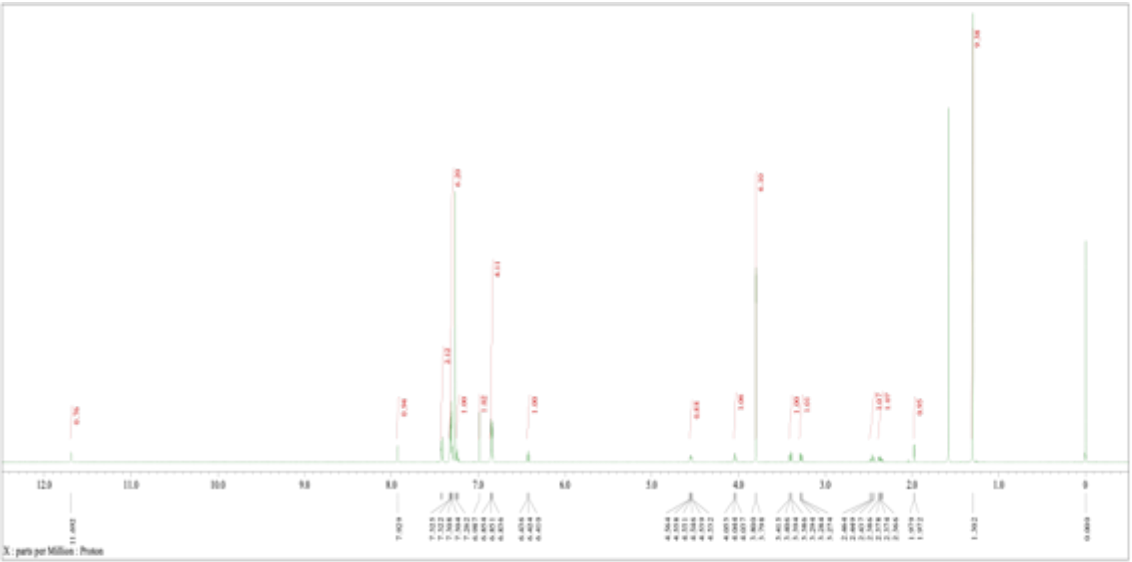

^{13}C NMR

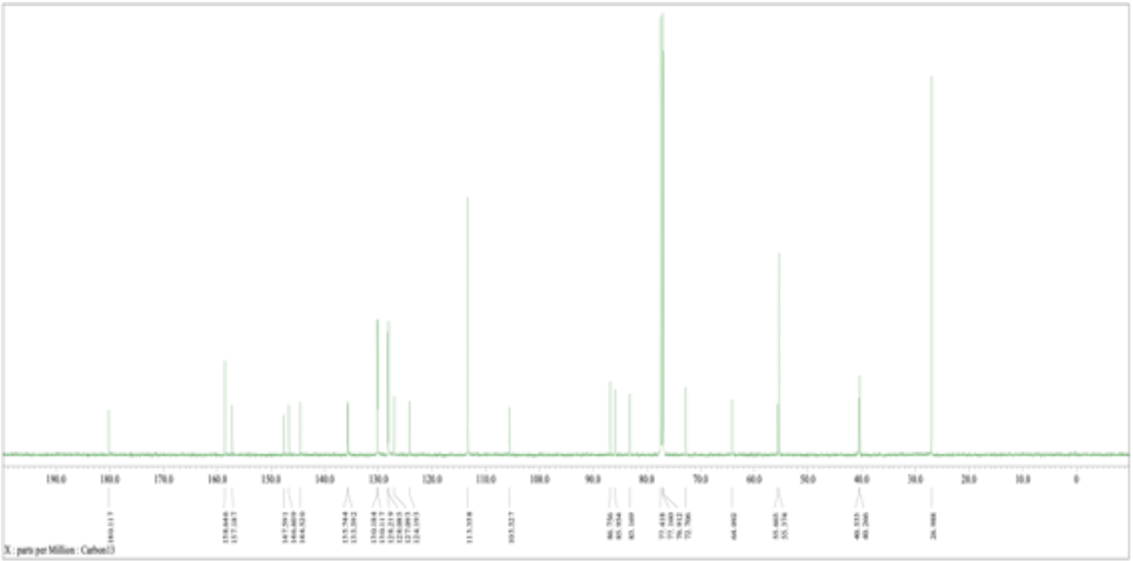

NMR spectra of compound 4

^{1}H NMR

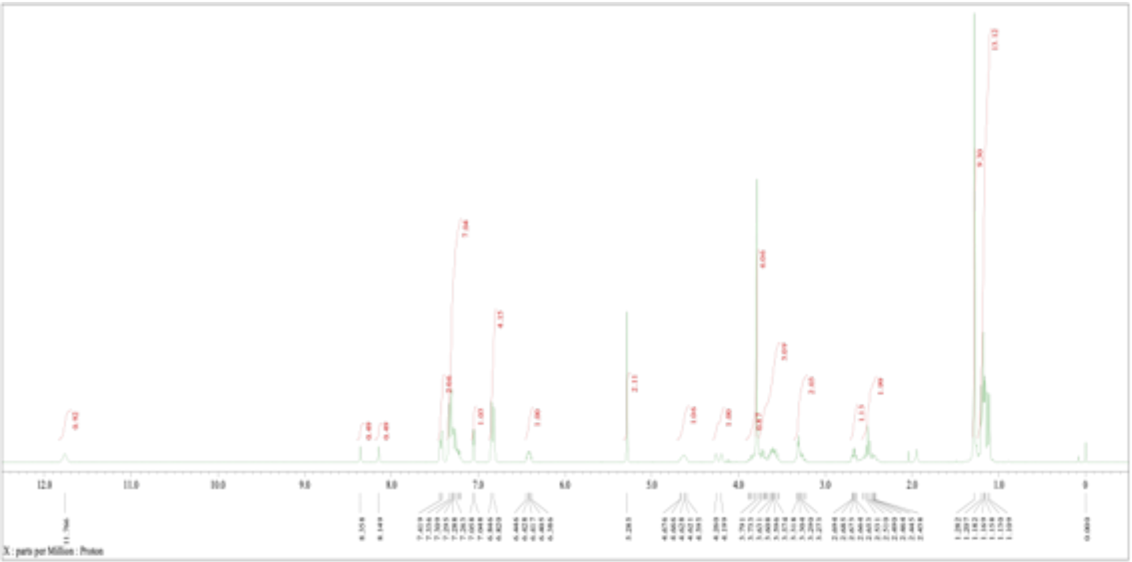

^{13}C NMR

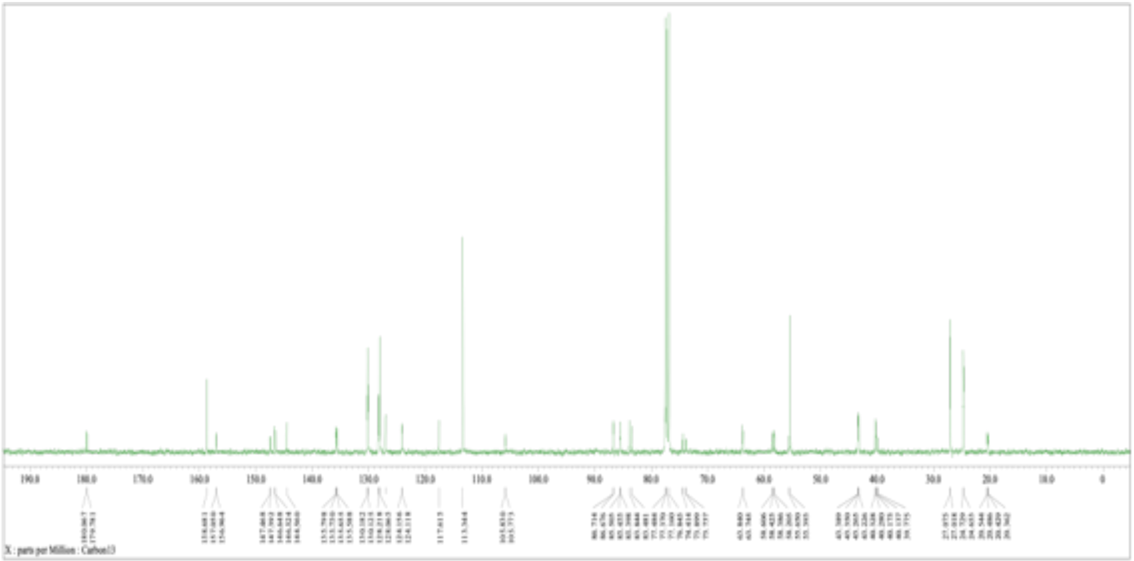

^{31}P NMR

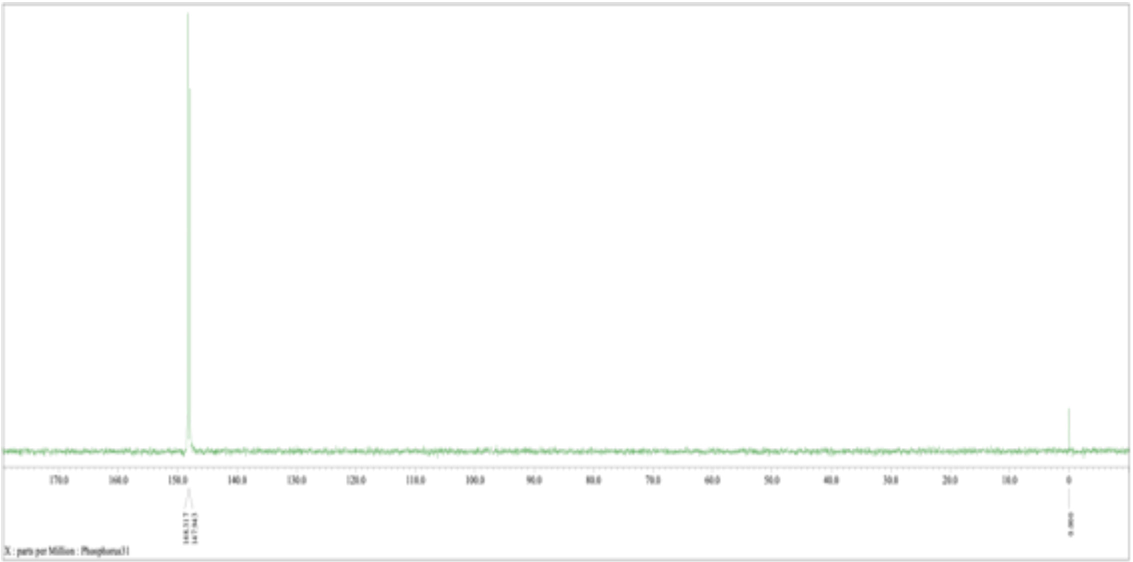

NMR spectra of compound 5

^{1}H NMR

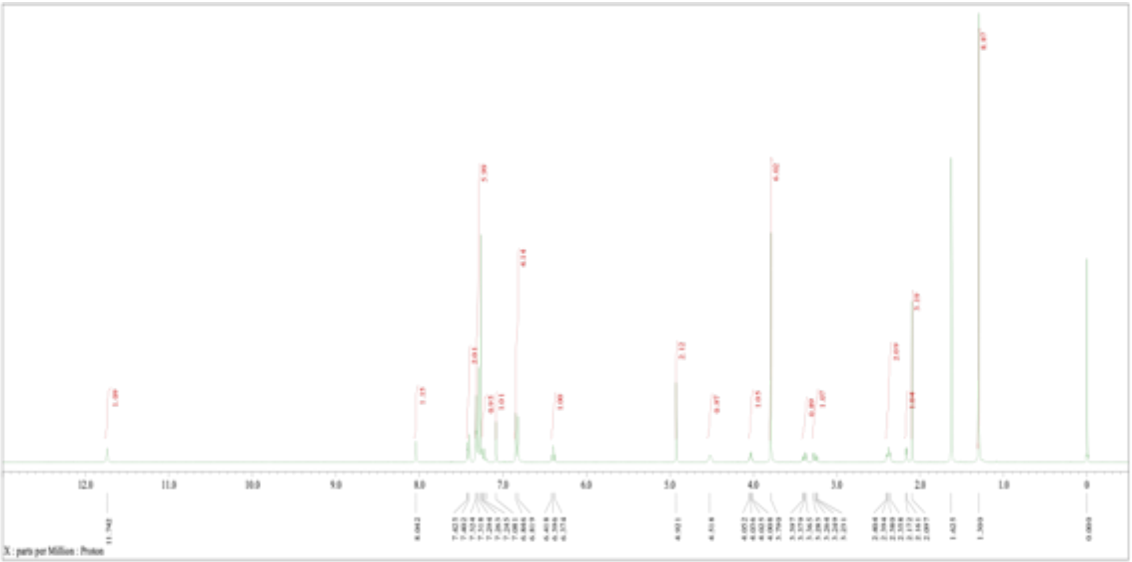

^{13}C NMR

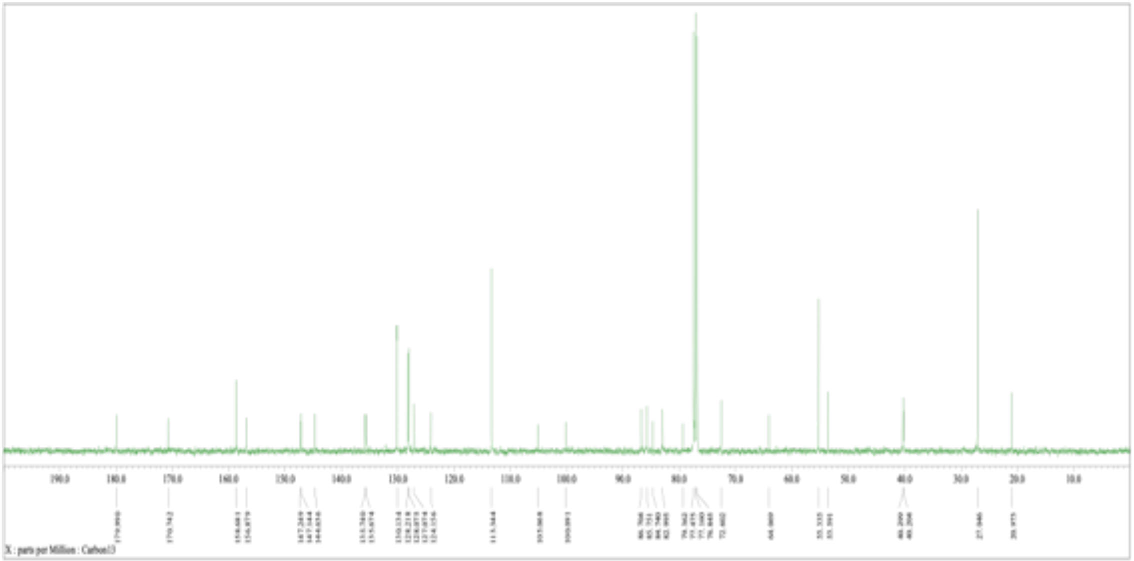

NMR spectra of compound 6

^{1}H NMR

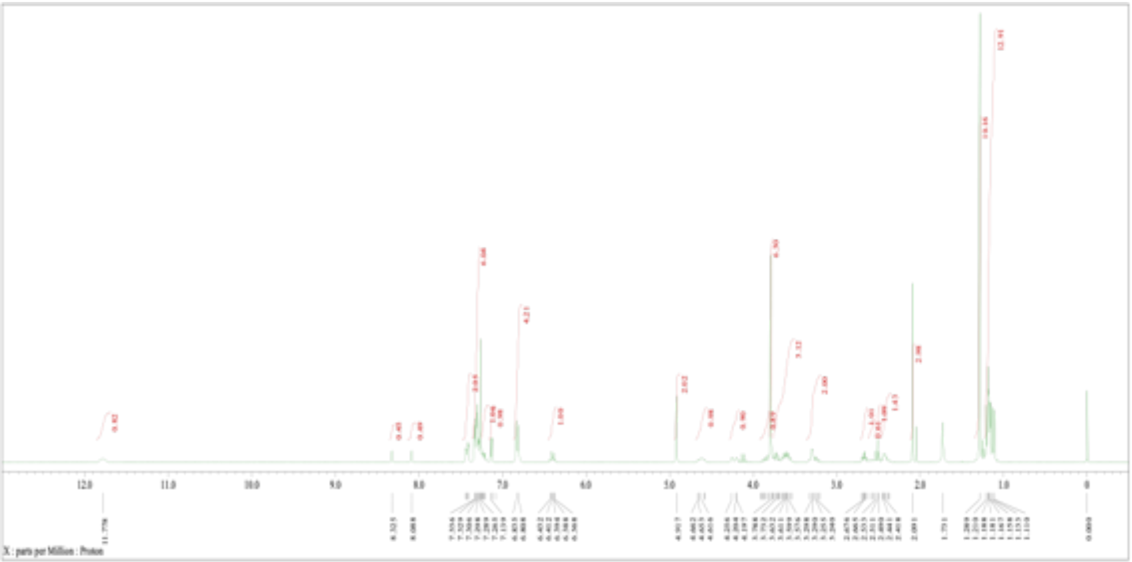

^{13}C NMR

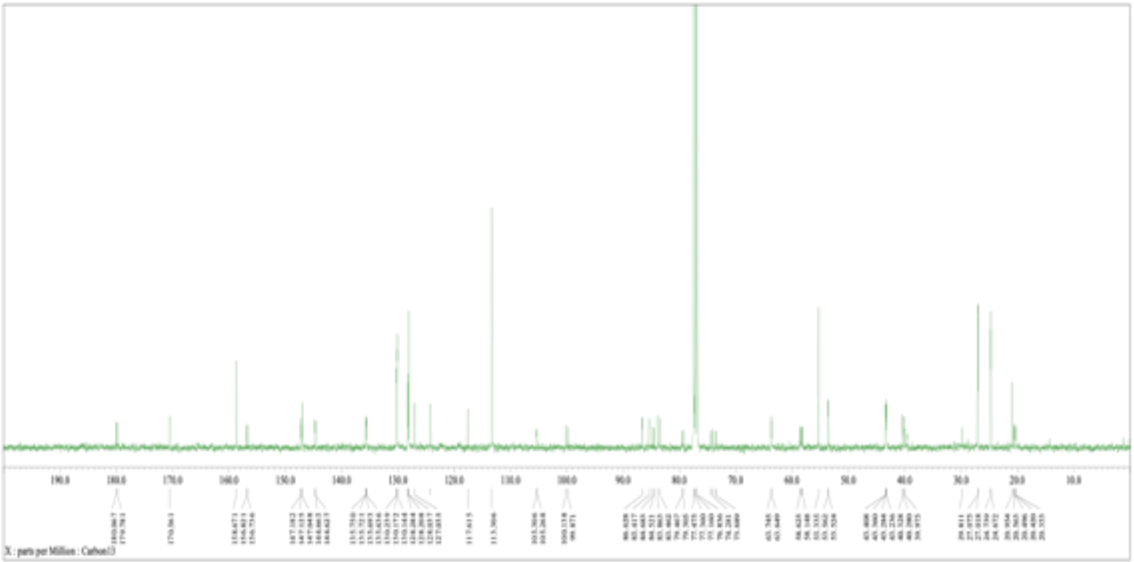

^{31}P NMR

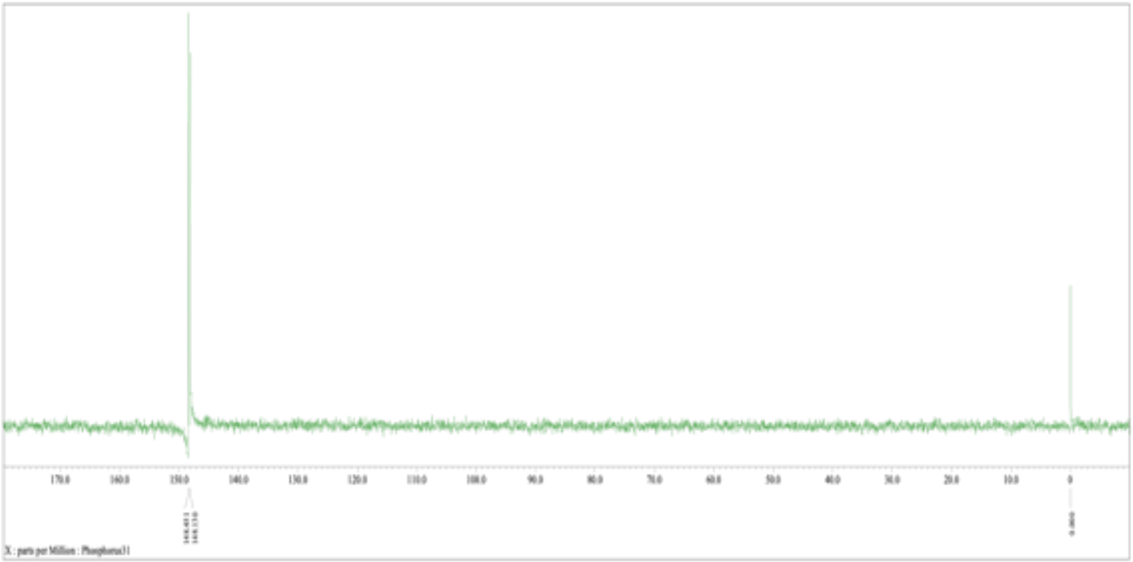
170.0 160.0 150.0 140.0 130.0 120.0 110.0 100.0 90.0 80.0 70.0 60.0 50.0 40.0 30.0 20.0 10.0 0
X : parts per Million : Phosphorus31

NMR spectra of compound 7

^{1}H NMR

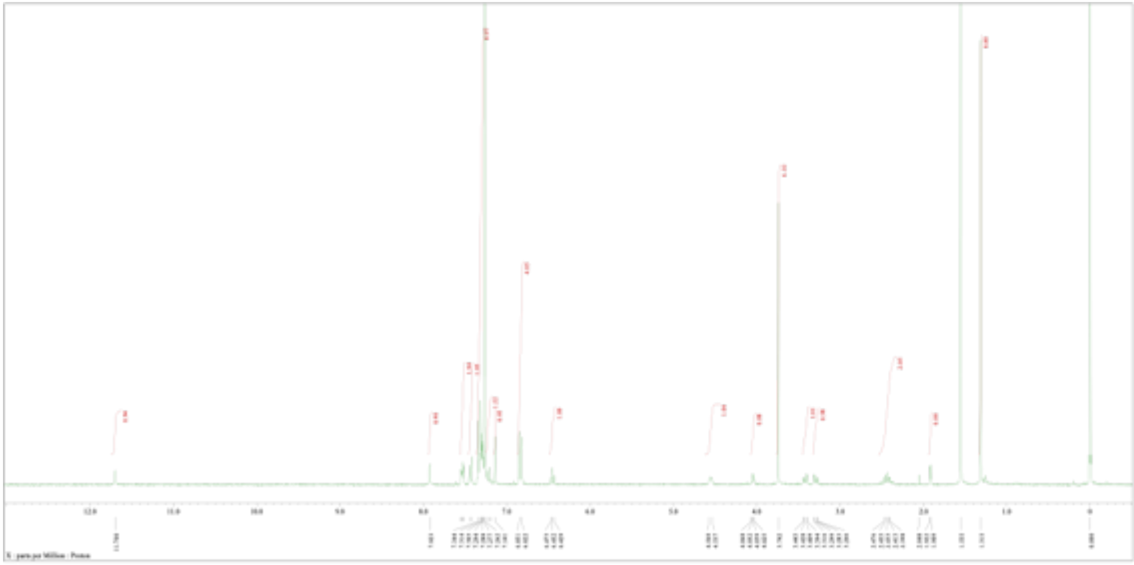

^{13}C NMR

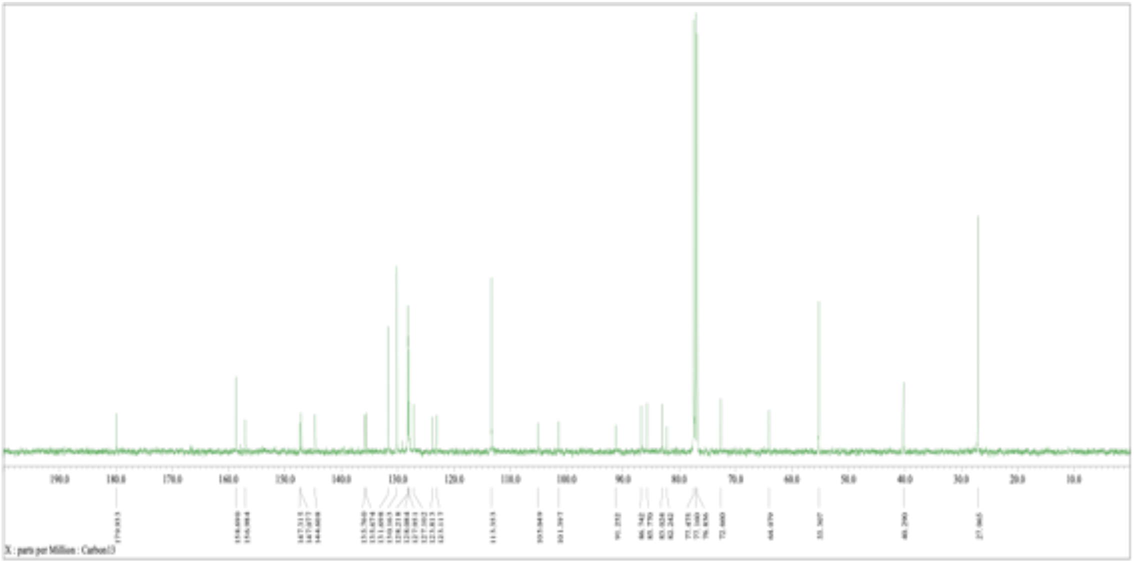

NMR spectra of compound 8

^{1}H NMR

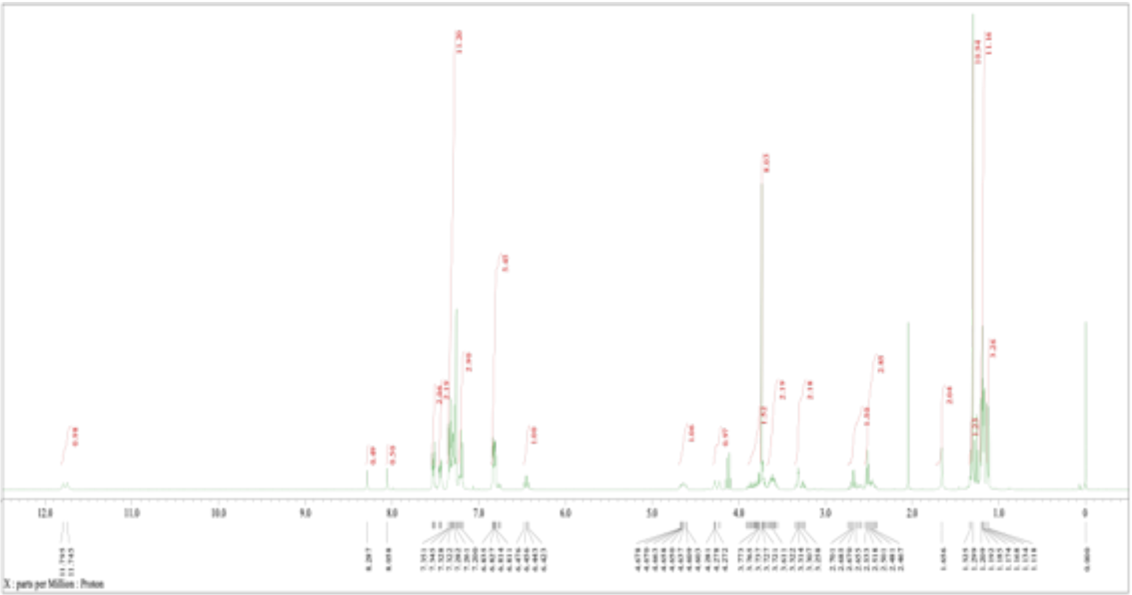

^{13}C NMR

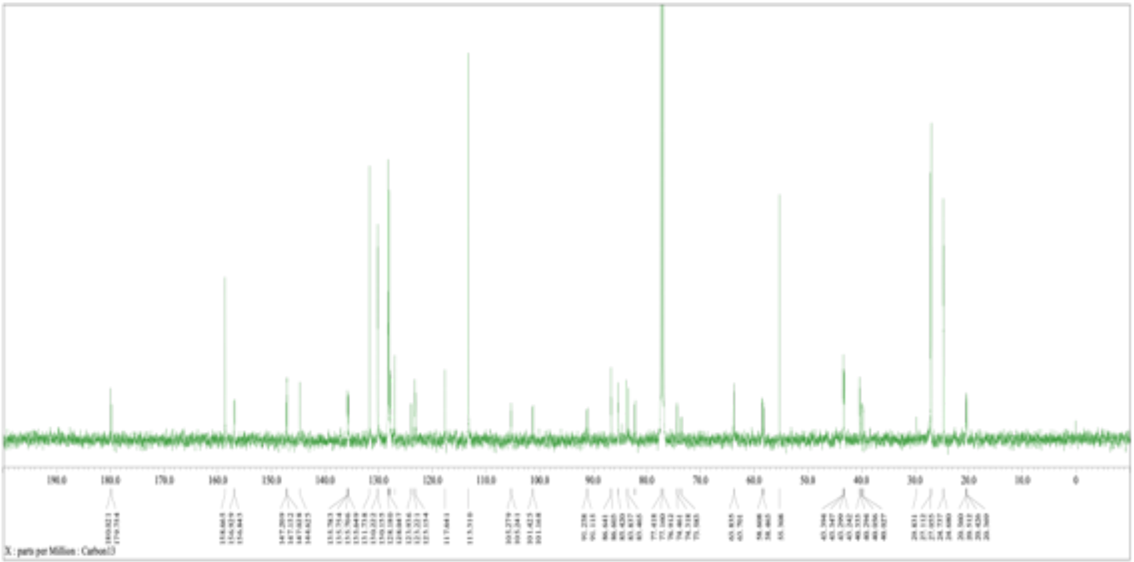

^{31}P NMR

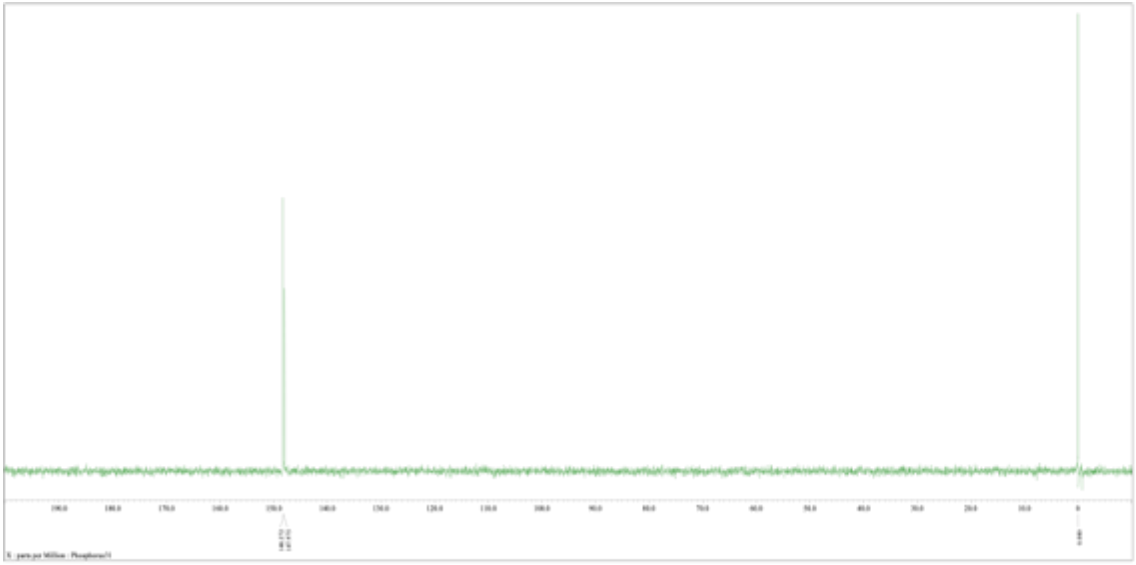

NMR spectra of compound 9

^{1}H NMR

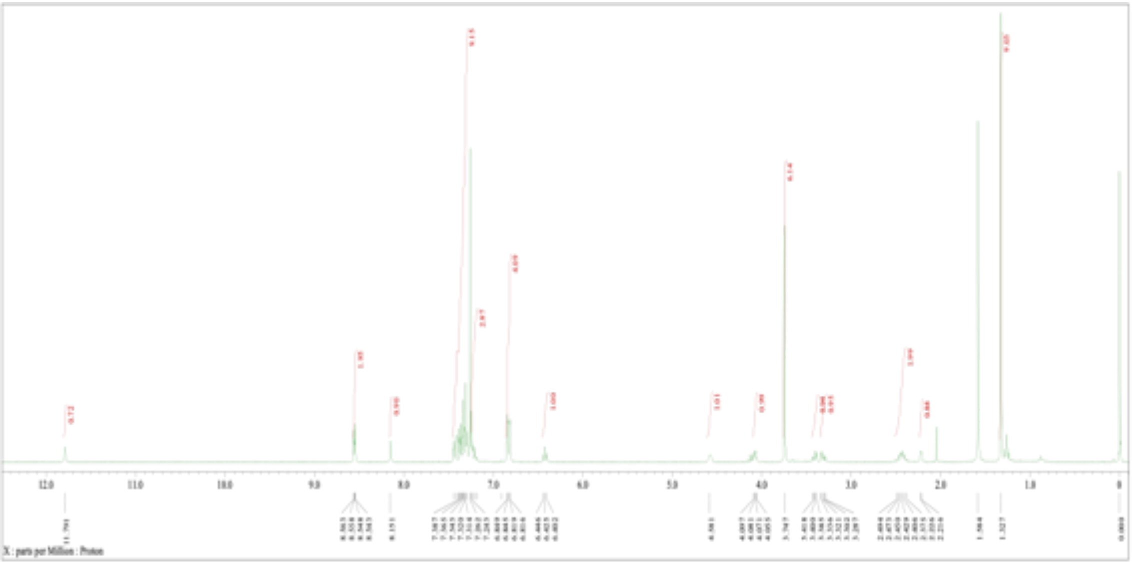

^{13}C NMR

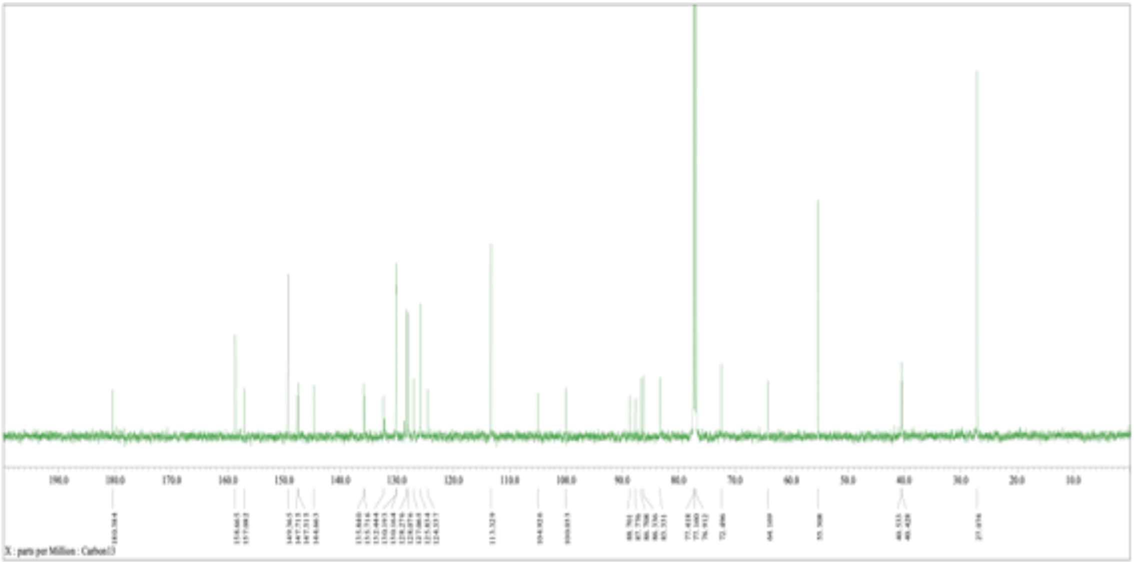

NMR spectra of compound 10

^1H NMR

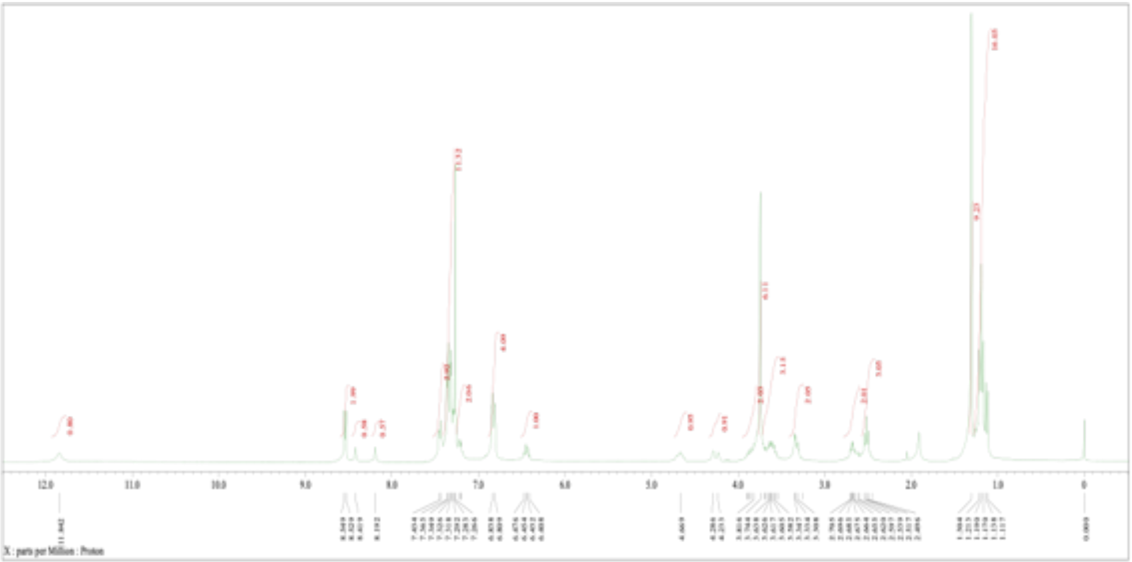

^{13}C NMR

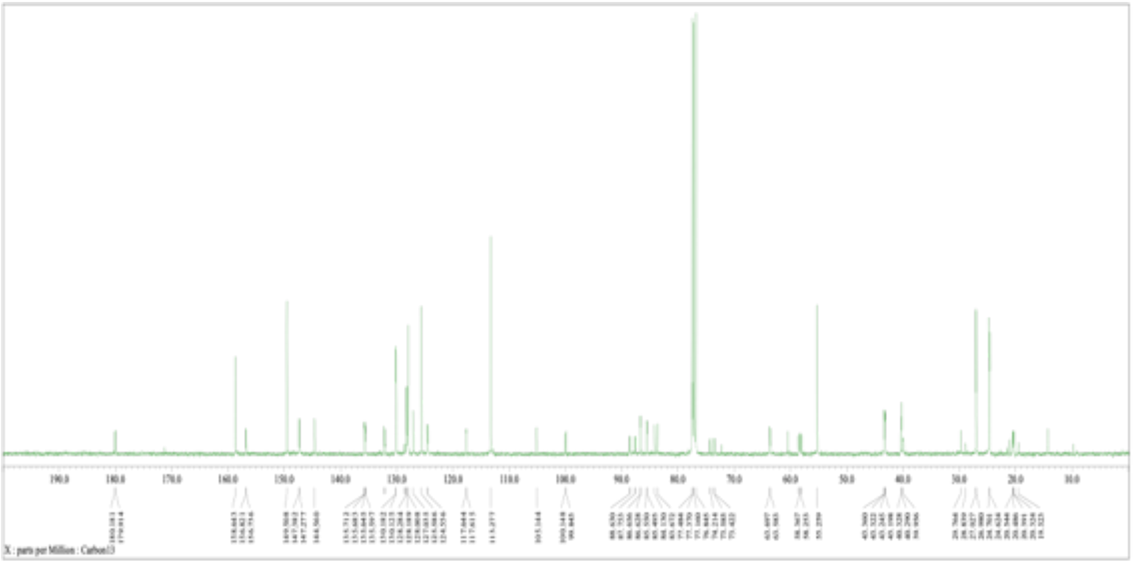

^{31}P NMR

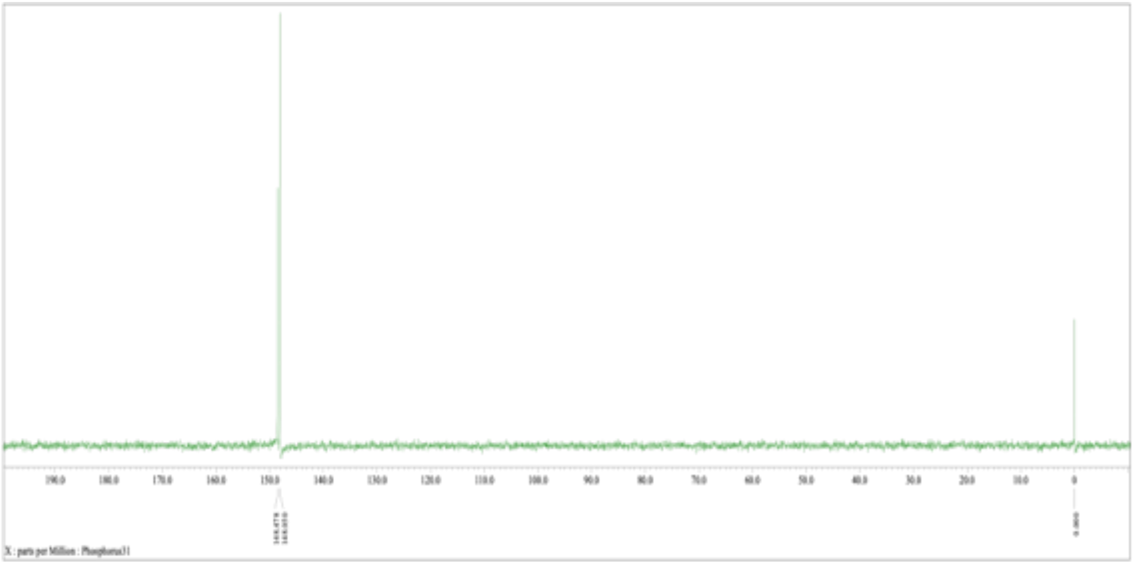

Supporting reference

1. Michaela Mačková, Soňa Boháčová, Pavla Perlíková, Lenka Poštová Slavětínská and Michal Hocek, *ChemBioChem* **2015**, *16*, 2225-2236.
